# Molecular characterization of colorectal cancer using whole‐exome sequencing in a Taiwanese population

**DOI:** 10.1002/cam4.2282

**Published:** 2019-05-24

**Authors:** Ya‐Sian Chang, Chien‐Chin Lee, Tao‐Wei Ke, Chieh‐Min Chang, Dy‐San Chao, Hsi‐Yuan Huang, Jan‐Gowth Chang

**Affiliations:** ^1^ Epigenome Research Center China Medical University Hospital Taichung Taiwan; ^2^ Department of Laboratory Medicine China Medical University Hospital Taichung Taiwan; ^3^ Center for Precision Medicine China Medical University Hospital Taichung Taiwan; ^4^ Department of Medical Laboratory Science and Biotechnology China Medical University Taichung Taiwan; ^5^ Department of Colorectal Surgery China Medical University Hospital Taichung Taiwan; ^6^ School of Medicine China Medical University Taichung Taiwan; ^7^ Department of Bioinformatics and Medical Engineering Asia University Taichung Taiwan

**Keywords:** colorectal cancer, gene mutation, next‐generation sequencing, pathway mutation

## Abstract

Next‐generation sequencing (NGS) technology is currently used to establish mutational profiles in many heterogeneous diseases. The aim of this study was to evaluate the mutational spectrum in Taiwanese patients with colorectal cancer (CRC) to help clinicians identify the best treatment method. Whole‐exome sequencing was conducted in 32 surgical tumor tissues from patients with CRC. DNA libraries were generated using the Illumina TruSeq DNA Exome, and sequencing was performed on the Illumina NextSeq 500 system. Variants were annotated and compared to those obtained from publicly available databases. The analysis revealed frequent mutations in *APC* (59.38%), *TP53* (50%), *RAS* (28.13%), *FBXW7* (18.75%), *RAF* (9.38%), *PIK3CA* (9.38%), *SMAD4* (9.38%), and *SOX9* (9.38%). A mutation in *TCF7L2* was also detected, but at lower frequencies. Two or more mutations were found in 22 (68.75%) samples.

The mutation rates for the WNT, P53, RTK‐RAS, TGF‐β, and PI3K pathways were 78.13%, 56.25%, 40.63%, 18.75%, and 15.63%, respectively. RTK‐RAS pathway mutations were correlated with tumor size (*P* = 0.028). We also discovered 23 novel mutations in *NRAS*, *PIK3CA*, *SOX9*, *APC*, *SMAD4*, *MSH3*, *MSH4*, *PMS1 PMS2*, *AXIN2*, *ERBB2*, *PIK3R1*, *TGFBR2*, and *ATM* that were not reported in the COSMIC, The Cancer Genome Atlas, and dbSNP databases. In summary, we report the mutational landscape of CRC in a Taiwanese population. NGS is a cost‐effective and time‐saving method, and we believe that NGS will help clinicians to treat CRC patients in the near future.

## 1 INTRODUCTION

Globally, colorectal cancer (CRC) is one of the most common human cancers and the fourth leading cause of cancer‐related death among males and females, with an estimated 1.4 million new cases and 694 000 deaths from the disease annually.^1^ In Taiwan, CRC ranked as the fourth leading cause of death, accounting for 14 965 cases diagnosed in 2012. CRC has increased significantly from 1990, with a growth rate of more than 2% per year worldwide. The likelihood of developing CRC is strongly correlated with old age, male gender, smoking, drinking alcohol, lack of exercise, being overweight, the consumption of red and/or processed meat, and a history of diabetes.^2^, ^3^

Epidermal growth factor receptor (EGFR) has been recognized as an effective anticancer target during the last few years. Monoclonal antibodies used to block EGFR in combination with chemotherapy or radiation have yielded improved outcomes in CRC patients with extended *RAS* wild‐type tumors. Mutations in the *RAS* and *BRAF* genes are harmful to anti‐EGFR therapy in metastatic CRC (mCRC).^4^ *RAS* and *BRAF* oncogene mutations are mutually exclusive and occur in 36.97% and 4.24% of CRC patients, respectively, as described in our previous work.^5^ Thus, identifying the unique genomic profiles and molecular phenotypes could help effectively establish the best treatment method in patients with anti‐EGFR therapy resistance.

CRC is one of the most interesting fields of next‐generation sequencing (NGS) application. The number of studies employing the NGS technique continues to increase. The Cancer Genome Atlas (TCGA) project studied more than 224 CRC cases and showed that 24 genes, including *APC*, *TP53*, *SMAD4*, *PIK3CA*, and *KRAS*, contained significant mutations. Three genes (*ARID1A*, *SOX9*, and *FAM123B/WTX*) were frequently mutated.^6^ Ashktorab et al analyzed 63 Iranian patients using targeted exome sequencing and found higher mutation rates of *MSH3*, *MSH6*, *APC*, and *PIK3CA* and hypothesized a larger role for these genes in CRC. They suggested the adoption of a specific informed genetic diagnostic protocol and tailored therapy in this population.^7^ Because patients with *RAS* wild‐type CRC can be non‐responders to EGFR‐targeted therapy, Geibler et al analyzed cell lines and tumor specimens to identify prediction markers by NGS, *EGFR* methylation and expression, and E‐cadherin expression. The authors revealed *ATM* mutations and low E‐cadherin expression as novel supportive predictive markers.^8^ Adua et al analyzed primary tumor and liver metastasis samples from 7 *KRAS* wild‐type patients and compared the genotypes of 22 genes associated with anti‐EGFR before and after chemotherapy. The results showed marked genotypic differences between pre‐ and post‐treatment samples, which were likely attributable to tumor cell clones selected by therapy.^9^ Gong et al analyzed 315 cancer‐related genes and introns of 28 frequently rearranged genes in 138 mCRC cases using FoundationOne. They identified a novel KRAS mutation (R68S) associated with an aggressive phenotype. The authors reported that *ERBB2*‐amplified tumors may benefit from anti‐HER2 therapy, and hypermutated tumors or tumors with high tumor mutational burden with MSI‐H or *POLE* mutation may benefit from anti‐PD‐1 therapy.^10^

This study examined genetic alterations in CRC in a Taiwanese population. We performed whole‐exome sequencing (WES) to detect the mutational status in all human protein‐coding genes using fresh frozen tissue from 32 Taiwanese patients with CRC.

## 2 MATERIALS AND METHODS

### 2.1 Study patients and tumor samples

This study was approved by the China Medical University Hospital Institutional Review Board. A summary of all patient characteristics is provided in Table 1. Patients ranged in age from 35 to 90 years, with a median age of 62 years. DNA was extracted using a QIAamp^®^ DNA Micro Kit (QIAGEN, Valencia, CA, USA) according to the manufacturer's instructions. Extracted DNA was immediately stored at −20°C until further processing. DNA concentration was measured by the Qubit dsDNA Assay Kit (Life Technologies, Carlsbad, CA, USA).

**Table 1:** Clinical features of 32 colorectal cancer patients

Characteristic	n (Frequency)
Age (years)	
Average: 60.47	Range: 35‐90
Sex	
Male	20
Female	12
Differentiation	
Low	2
Middle	28
Middle to Low	2
AJCC stage	
I	4
IIA	15
IIIB	5
IIIC	2
IVA	1
IVB	4
NA	1
Regional lymph node metastasis	
N0	19
N1	4
N2	7
NA	2
Site	
Rectum	8
Colon	24

### 2.2 WES and data analysis

DNA libraries were prepared using the Illumina TruSeq Exome Library Prep Kit and sequenced on the Illumina NextSeq 500 platform. Base calling and quality scoring were performed by an updated implementation of Real‐Time Analysis in NextSeq500. Bcl2fastq Conversion Software was used to demultiplex data and convert BCL files into FASTQ files. Sequenced reads were trimmed for low‐quality sequences and aligned to the human reference genome (hg 19) using Burrows‐Wheeler Alignment.^11^ Finally, single nucleotide polymorphisms and small insertion and deletion mutations were called in individual samples by the Genome Analysis Toolkit and VarScan using default settings.^12^, ^13^ We then performed ANNOVAR to functionally annotate genetic variants.^14^ The following criteria were used to select confident somatic single nucleotide variants: mutant allele frequency >5%, global minor allele frequency <1%, or NA (comparing the ExAC and 1000 Genome Databases data), eliminating known harmless variants present in ClinVar or the in‐house polymorphism database, and predicted to be pathogenic by all three software programs (SIFT, PolyPhen‐2, and CADD).

### 2.3 Statistical analysis

Comparisons between clinicopathological features and the status of critical pathway mutations in CRC were performed using Fisher's exact test. Two‐sided *P*‐values < 0.05 were considered statistically significant.

## 3 RESULTS

### 3.1 WES analysis and coverage

**Figure 1:**
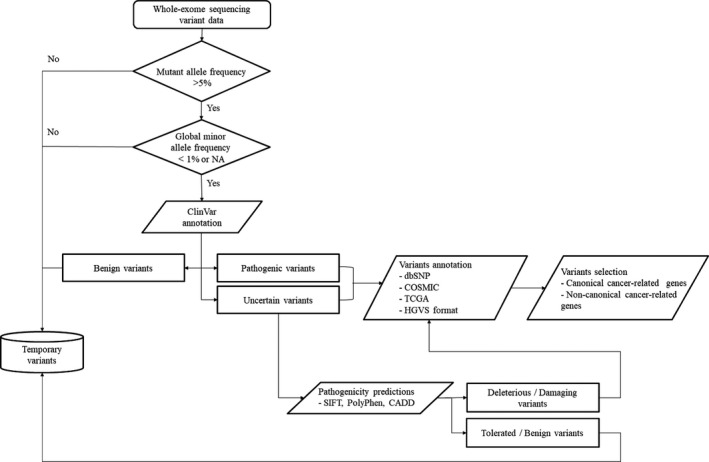
Overview of our approach used to identify variants

Using massive parallel sequencing on a NextSeq platform, we generated a mean of 157 M raw reads per sample, of which 141 M were aligned to the human reference genome (hg19; Table 2). The mean depth of the target regions for the 32 samples was 119× (range 34.79‐197.53×). The coverage of the target regions exceeded 97.97%. Figure 1 is an overview of our approach used to identifying variants.

**Table 2:** Alignment and coverage statistics for 32 colorectal cancer patients

Patient ID	Total raw reads	Total effective reads	Reads mapped to genome	Average sequencing depth on target	Coverage on target (%)
16	82 864 708	66 661 376	66 657 979	47.14	98.44
25	69 110 948	56 550 852	56 544 621	40.16	98.00
36	68 965 280	56 546 730	56 539 081	38.51	98.10
50	356 294 022	326 553 966	326480947	188.98	99.01
56	75 141 654	60 803 428	60 794 779	43.41	97.97
62	71 243 396	58 278 776	58 270 698	40.07	98.12
71	70 086 092	57 388 574	57 381 580	41.24	98.05
89	63 437 554	50 916 024	50 913 067	37.87	98.35
93	59 001 856	47 743 596	47 736 346	34.79	98.03
98	269 310 102	24 7274 550	247 189 402	197.53	99.37
99	63 078 404	51 065 200	51 058 021	37.07	98.10
103	66 173 134	52 911 932	52 907 746	39.03	98.31
CC01	202 308 880	182 302 644	182 249 487	148.28	98.93
CC02	196 162 260	179 076 152	179036670	137.71	98.86
CC03	149 966 094	138 301 996	138 263 040	124.07	99.16
CC04	188 762 344	175 316 188	175 287 324	154.65	99.17
CC05	174 170 480	161 466 102	161 439 317	143.79	98.92
CC06	163 747 730	151 903 466	151 881 413	128.67	99.13
CC07	180 821 438	167 186 452	167 155 256	133.86	99.00
CC08	174 412 902	161 772 158	161 747 641	146.76	99.17
CC10	178 559 504	160 173 326	160 136 434	148.31	99.15
CC11	202 264 106	182 800 322	182 757 243	168.55	98.94
CC12	203 133 950	183 665 658	183 629 660	164.16	98.92
CC13	195 342 238	176 816 668	176 779 527	163.11	99.14
CC14	215 392 940	192 504 740	192 468 467	176.64	98.96
CC15	186 503 740	168 736 670	168 699 555	150.49	99.13
CC16	188 775 628	173 447 948	173 418 659	160.24	99.20
CC17	189 597 468	174 502 714	174 458 692	157.42	99.21
CC18	179 218 892	164 454 320	164 426 639	153.55	98.96
CC20	179 435 082	165 011 368	164 988 404	155.39	98.95
CC21	195 883 102	179 886 726	179 858 958	168.34	99.21
CC24	173 198 708	159 597 160	159 569 988	153.26	98.92
Average	157 261 395	141 613 056	141 585 208	119.47	98.78

### 3.2 CRC‐associated oncogene variants

#### 3.2.1 *RAS* mutations

**Figure 2:**
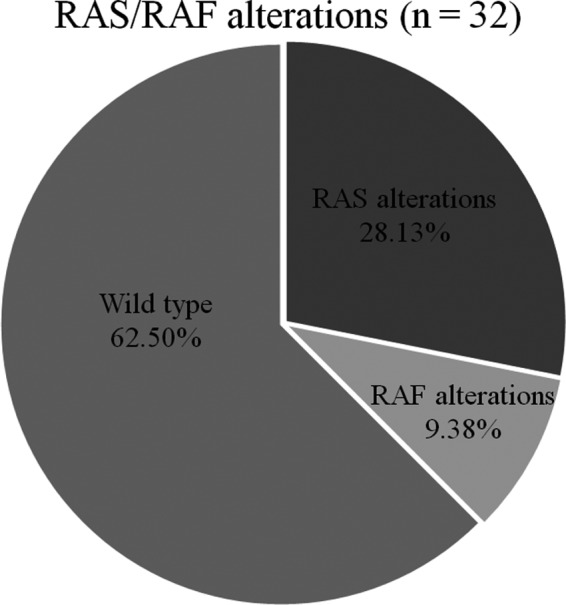
Proportion of *RAS*, *RAF* mutations, and *RAS*/*RAF* wild‐type status identified by WES. WES, whole‐exome sequencing

**Figure 3:**
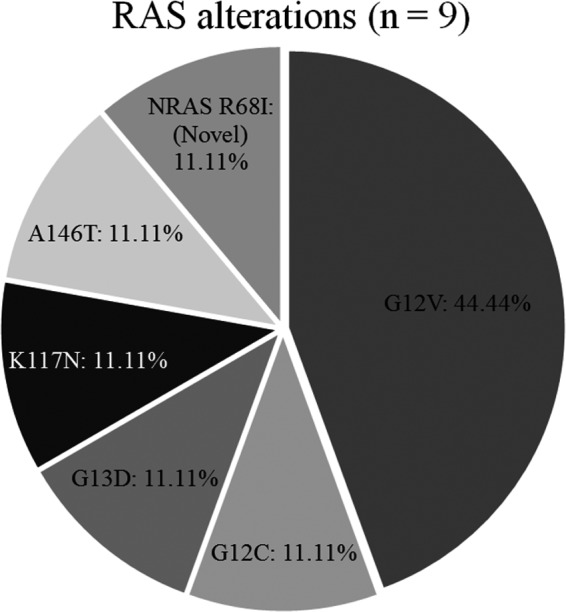
Proportion of *RAS* alterations identified by WES. WES, whole‐exome sequencing

Overall, *RAS* mutations were present in 28.13% of our CRC patients (Figure 2). The most common *RAS* mutations were *KRAS* mutations in exon 2 (codons 12 and 13), including G12V (44.44%), G12C (11.11%), and G13D (11.11%). Beyond the well‐established point mutations in codons 12 and 13 of exon 2 of *KRAS*, we identified mutations in codon 117 of exon 4 (K117N, 11.11%) and codon 146 of exon 4 (A146T, 11.11%). One mutation (11.11%) in codon 68 (exon 3) of *NRAS* was also detected; this was a novel alteration (R68I). The non‐synonymous variant at locus 115256508 had a C‐to‐A change mapped in the small GTP‐binding protein domain, with an allele fraction of 21.19% (total reads 118, variant count 25) (Figure S1A). Together, these non‐*KRAS* exon 2 mutations constituted 33.33% of all *RAS* mutations (Figure 3).

#### 3.2.2 *RAF* mutations

Two *RAF* mutations were found in 9.38% of our patients (Figure 2). Two patients (6.25%) had *BRAF* V600E mutations. One patient (3.13%) had an *ARAF* T256fs mutation. None of the CRC patients with *RAS* mutations harbored a concomitant mutation in *RAF*. The remaining patients (62.5%) were *RAS*/*RAF* wild‐type (Figure 2).

#### 3.2.3 *PIK3CA* mutations

Three patients (9.38%) had *PIK3CA* mutation tumors. The mutation variants were R38S, G118D, and D350Y; D350Y was a novel mutation. The non‐synonymous variant at locus 178921566 had a G‐to‐T change mapped in the phosphatidylinositol 3‐kinase, C2 domain, with an allele fraction of 17.53% (total reads 97, variant count 17) (Figure S1B).

#### 3.2.4 *TCF7L2* mutations

Two patients (6.25%) had *TCF7L2* mutation tumors. The identified variants were R471C, F357L, and G424E, and each patient had two of the three *TCF7L2* variants.

#### 3.2.5 *SOX9* mutations

Three patients (9.38%) had *SOX9* frameshift mutations. One patient had an S431fs mutation, another a G484fs mutation, and the third an S485fs mutation. The G484fs and S485fs mutations were novel variants (Figure S1C).

### 3.3 CRC‐associated tumor suppressor gene variants

#### 3.3.1 *APC* mutations

In total, we identified 19 patients (59.38%) with *APC* alterations. A total of 26 *APC* mutations were identified in the 19 samples, most of which were nonsense mutations that introduced a premature stop codon (R283*, S320*, Q541*, R564*, R876*, R1114*, Q1294*, E1309*, Q1367*, Q1378*, R1450*, E1544*, Q1916*, and R2204*). Six variants were frameshift deletions (L620fs, D1297fs, E1306fs, G1312fs, E1374fs, and E1397fs), 5 were frameshift insertions (L540fs, L852fs, T1292fs, L1302fs, and E1554fs,), and 1 was a missense mutation (S1400L). Among these mutations, 7 novel mutations were found (L540fs, T1292fs, D1297fs, L1302fs, E1306fs, E1374fs, and Q1916*) (Figure S1D).

#### 3.3.2 *TP53* mutations

Overall, *TP53* mutations were present in 50% of our CRC patients. Fifteen *TP53* mutations were identified in the 16 samples. All variants have been reported (L43fs, K132N, P151S, R175H, C176F, R196*, L206*, M237I, R245C, M246R, E258K, R273H, R273C, R282W, and R306*).

#### 3.3.3 *FBXW7* mutations

Six of the 32 samples (18.75%) had a mutation in *FBXW7*. Four *FBXW7* variants were found in the 6 samples. All variants have been reported (G80W, W307C, R347H, and R387C).

#### 3.3.4 *SMAD4* mutations

Three patients (9.38%) had *SMAD4* mutations. Two variants have been reported previously (G419R and R496H), and the other was novel (Y260_H261delins*). The frameshift variant at locus 48584605 had an A insertion with an allele fraction of 22.18% (total reads 284, variant count 63) (Figure S1E).

### 3.4 Mismatch repair (MMR) gene variants

#### 3.4.1 *MLH1, MSH3, MSH4*, *PMS1*, and *PMS2* mutations

Five patients (15.63%) had mismatch repair (MMR) gene mutations. Mutations in the MMR gene included *MLH1*, *MSH3, MSH4*, *PMS1*, and *PMS2*. The mutation variants were R385C and T117M in *MLH1*, A61delinsAAPA and E456K in *MSH3*, E583* in *MSH4*, R265Q in *PMS1*, and L633I in *PMS2*. Among these, *MSH3* A61delinsAAPA and E456K, *MSH4* E583*, *PMS1* R265Q, and *PMS2* L633I were novel mutations (Figure S1F‐I). The numbers of variants discovered in the MMR wild‐type and mutation carriers are listed in Tables S1 and S2.

### 3.5 Altered signaling pathways in CRC

**Figure 4:**
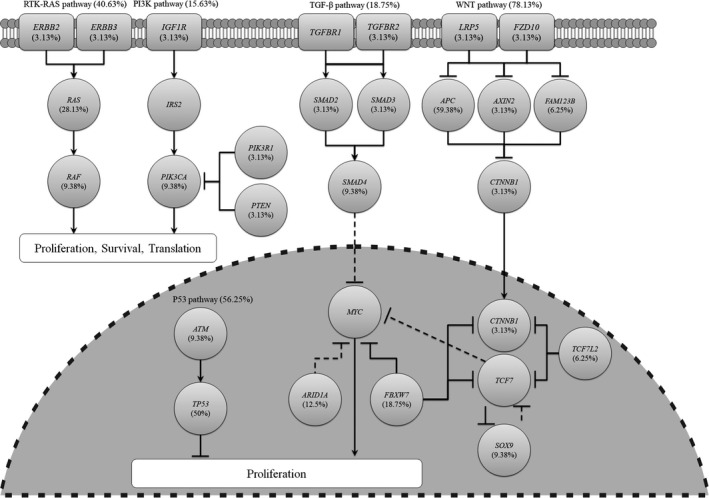
Frequency of genetic changes leading to deregulation of signaling pathways in CRC. CRC, colorectal cancer

Based on our analytical approach, we identified multiple genes in the RTK‐RAS, PI3K, TGF‐β, WNT, and P53 pathways. The *APC* gene in the WNT pathway had relatively high levels of somatic mutations compared to genes in the RTK‐RAS, PI3K, TGF‐β, and P53 pathways. We found 10 different altered WNT pathway genes, including *LRP5*, *FZD10*, *APC*, *AXIN2*, *FAM123B*, *CTNNB1*, *TCF7L2*, *SOX9*, *FBXW7*, and *ARID1A*, confirming the importance of this pathway in CRC. We found that 78.13% of tumors had alterations in the WNT pathway. We also evaluated genetic alterations in the RTK‐RAS, PI3K, TGF‐β, and P53 pathways, with mutation rates of 40.63%, 15.63%, 18.75%, and 56.25%, respectively (Figure 4).

### 3.6 Pathway mutations and associations

We compared the clinicopathological data of CRC patients with mutations in mutation‐related pathways. The RTK‐RAS pathway mutation rate was significantly higher in patients with a tumor size ≤4 cm compared to those with a tumor of >4 cm (57.89% versus 15.38%, *P* = 0.028). No clinicopathological variables were significantly correlated with WNT, PI3K, TGF‐β, or P53 pathway mutations (Table 3).

**Table 3:** Correlation between clinicopathological features and mutational status

		Mutation of WNT pathway				Mutation of RTK‐RAS pathway				Mutation of PI3K pathway				Mutation of TGF‐β pathway				Mutation of P53 pathway			
		No	Yes	Total	*P*‐Value	No	Yes	Total	*P*‐Value	No	Yes	Total	*P*‐Value	No	Yes	Total	*P*‐Value	No	Yes	Total	*P*‐Value
Gender	F	3	9	12	1.000	7	5	12	1.000	10	2	12	1.000	11	1	12	0.370	4	8	12	0.471
	M	4	16	20		12	8	20		17	3	20		15	5	20		10	10	20	
Age	<62	3	13	16	1.000	10	6	16	1.000	15	1	16	0.333	13	3	16	1.000	5	11	16	0.285
	≥62	4	12	16		9	7	16		12	4	16		13	3	16		9	7	16	
Tumor Size	≤4 cm	3	16	19	0.401	8	11	19	0.028	17	2	19	0.375	15	4	19	1.000	10	9	19	0.289
	>4 cm	4	9	13		11	2	13		10	3	13		11	2	13		4	9	13	
Stage	I, II	3	16	19	0.384	12	7	19	1.000	15	4	19	0.624	16	3	19	0.653	7	12	19	0.710
	III, IV	4	8	12		7	5	12		11	1	12		9	3	12		6	6	12	
Site	Rectum	3	5	8	0.327	5	3	8	1.000	6	2	8	0.578	8	0	8	0.296	4	4	8	0.704
	Colon	4	20	24		14	10	24		21	3	24		18	6	24		10	14	24	
LN metastasis	−	3	16	19	0.401	12	7	19	0.720	15	4	19	0.625	16	3	19	0.666	7	12	19	0.473
	+	4	9	13		7	6	13		12	1	13		10	3	13		7	6	13	

*P*‐Value by Fisher's Exact Test.

## 4 DISCUSSION

All of the mutated genes discussed in our study have been previously classified as driver genes that confer a selective growth advantage to tumor cells harboring the mutations. CRC is similar to other cancers with only one or multiple driver gene mutations. Tumors with only one driver mutation, always in an oncogene, and with multiple driver mutations contain a combination of oncogene and tumor suppressor gene mutations.^15^ In our study, of the 4 samples with a single mutation (Table 4), 1 (25%) harbored a mutation in an oncogene (*KRAS*), and of the 22 samples with 2 or more mutations (Tables 5 and 6), 15 (68.18%) contained a combination of mutations in both oncogenes and tumor suppressor genes.

**Table 4:** Single point mutations detected in 32 colorectal cancer samples

Genes	Mutation	Sex	Age (years)	Differentiation	AJCC stage
*TP53*	p.K132N	F	57	Middle	IVB
*APC*	p.Q1294*	M	57	Middle	IIIB
*MSH3*	p.A61delinsAAPA	M	61	Middle	IIA
*KRAS*	p.G12C	F	69	Middle	IIA

**Table 5:** Double combination mutations detected in 32 colorectal cancer samples

Gene 1	Mutation 1	Gene 2	Mutation 2	Sex	Age (years)	Differentiation	AJCC stage
*ARAF*	p.T256fs	*FBXW7*	p.W307C	M	65	Middle to Low	NA
*APC*	p.Q1916*	*MLH1*	p.T117M	M	72	Middle	IIA
*KRAS*	p.G12V	*TP53*	p.C176F	F	57	Middle	IIIB
*APC*	p.Q1367*	*TP53*	p.R282W	M	61	Middle	IIIB
*SOX9*	p.G485fs	*APC*	p.R283*	F	78	Middle	IIA
*APC*	p.L540fs p.R1450*	*TP53*	p.L43fs	M	35	Middle	IIA
*KRAS*	p.G12V	*APC*	p.R564* p.L1302fs	M	68	Middle	IIA
*APC*	p.Q541*	*TP53*	p.M246R	F	58	Middle	IIA
*APC*	p.L620fs p.E1306fs	*TP53*	p.L206*	F	47	Middle	I
*APC*	p.S320* p.E1544*	*FBXW7*	p.R387C	F	42	Middle	IIIB

**Table 6:** Three or more combination mutations detected in 32 colorectal cancer samples

Gene 1	Mut. 1	Gene 2	Mut. 2	Gene 3	Mut. 3	Gene 4	Mut. 4	Gene 5	Mut.5	Gene 6	Mut. 6	Gene 7	Mut. 7	Sex	Age	Diff	AJCC stage
*BRAF*	p.V600E	*TP53*	p.E258K	*FBXW7*	p.G80W									M	55	Low	IVB
*KRAS*	p.G12V	*APC*	p.E1554fs	*TP53*	p.R175H									F	63	M	IIA
*SOX9*	p.S431fs	*APC*	p.L852fs p.T1292fs	*TP53*	p.R196*									M	63	M	
*KRAS*	p.G13D	*APC*	p.Q1378*	*FBXW7*	p.R347H									M	63	M	I
*APC*	p.E1374fs	*TP53*	p.R273C	*SMAD4*	p.R496H									M	68	M	IIA
*KRAS*	p.A146T	*APC*	p.G1312fs	*TP53*	p.P151S	*MLH1*	p.R385C							F	45	M	IIA
*NRAS*	p.R68I	*APC*	p.E1397fs	*TP53*	p.R245C p.R282W	*FBXW7*	p.R347H							M	48	M	I
*PIK3CA*	P.G118D	*APC*	p.D1297fs	*FBXW7*	p.R387C	*TP53*	p.R273H							F	67	M	IIA
*KRAS*	p.G12V	*TP53*	p.R273C	*SMAD4*	p.G419R	SOX9	p.S484fs							F	64	L	IIA
*BRAF*	p.V600E	*APC*	p.Q1294* p.E1554fs	*TP53*	p.M237I	*SMAD4*	p.Y260_H261delins*							M	44	M	IVB
*KRAS*	p.K117N	*PIK3CA*	p.R38S	*TCF7L2*	p.R471C p.G424E	*APC*	p.R876* p.E1309* p.R2204*	*MSH4*	p.E583*					M	71	M to L	IIIC
*PIK3CA*	p.D350Y	*TCF7L2*	p.F357L p.R471C	*APC*	p.R1114* p.Q1378* p.S1400L	*TP53*	p.R306*	*MSH3*	p.E456K	*PMS1*	p.R265Q	*PMS2*	p.L633I	M	50	M	IIA

The integrative analysis of WES data provides insights into pathways that are dysregulated in CRC. The WNT signaling pathway was dysregulated in 78.13% of cases. WNT pathway mutations have been reported in 84.5%% of CRC cases, which is higher than the mutation rate detected in our study.^16^ In 2012, the TCGA consortium reported that up to 93% of CRC cases involved at least 1 alteration in a known WNT regulator.^6^ Hyperactivation of the WNT pathway initiates the development of CRC, which predominantly occurs through inactivation of the *APC* gene.^17^ Several agents have been investigated to target this pathway, including WNT inhibitors (eg, Rofecoxib, PRI‐724, CWP232291) and a monoclonal antibody against frizzled receptors (e.g., vanituctumab).^18^ In addition to *APC* and *SOX9*, we also identified a novel mutation in *AXIN2* (p.R459L) (Figure S1J). The *AXIN2* mutation identified in the current study, R459, is located in the region that interacts with β‐catenin.

The frequency of alterations in the RTK‐RAS and PI3K pathways was 40.63% and 15.63%, respectively. RTK‐RAS and PI3K pathway mutations have been found in 60.7% and 30% of CRCs, respectively.^16^ In a normal cell, RTK‐RAS and PI3K pathways control cell proliferation, differentiation, and survival.^19^, ^20^ In a malignant cell, constitutive and aberrant activation of components of these pathways lead to increased cell growth, survival, and metastasis. Small molecule inhibitors, such as Sorafenib and PLX4720, which are currently being used to target BRAF p.V600E, have been developed to target the RTK‐RAS and PI3K pathways. NVP‐BEZ235 and BGT226 are being used to target the PI3K pathway in various cancers.^21^ In addition to *NRAS* and *PIK3CA*, we identified two novel mutations in *ERBB2* (p.W9fs) and *PIK3R1* (p.S147* and p.L161*) (Figure S1K,L). The *PIK3R1* p.S147* and p.L161* mutations were mapped to the Rho GTPase‐activating protein domain.

In our study population, the mutation rate of the TGF‐β and P53 pathways was 18.75% and 56.25%, respectively. TGF‐β and P53 pathway mutations have been described in 28.9% and 69% of CRCs, respectively.^16^ The TGF‐β signaling pathway has pleiotropic functions, including the regulation of cell growth, apoptosis, cell motility, and invasion. TGF‐β signaling plays a key role in tumor initiation, development, and metastasis. Many TGF‐β pathway inhibitors, such as antisense oligonucleotides, neutralizing antibodies, and receptor kinase inhibitors, have been used in preclinical trials. For example, galunisertib is a TGFβR1 inhibitor that prevents signal transduction.^22^ Under cellular stress, such as DNA damage, oncogenes, oxidative free radicals, and UV irradiation, the P53 protein is activated. Activation of P53 can induce cell cycle arrest, senescence, and apoptosis. Small molecular inhibitors, such as MIs, nutlins, and RITA, have been tested as therapeutic agents in CRC by activating this pathway.^23^ In addition to *SMAD4*, we identified a novel mutation in *TGFBR2* (p.D549A) and *ATM* (p.E650*) (Figure S1M,N). Our relatively low rate of mutations in these 5 critical pathways may reflect our small sample size.

Most CRC samples can be grouped by WNT‐, RTK‐RAS‐, P53‐, TGF‐β‐, and PI3K‐dysregulated pathways. In our study population, 3 samples (3/32, 9.38%) had no mutation in any of these pathways. However, in these 3 samples, 2 had alterations in the Notch signaling pathway (*CTBP2*, *CREBBP*, *KAT2B*, *DVL2*, and *PSEN2*). Deregulation of Notch signaling in CRC has been reported.^24^ The third sample exhibited alterations in cell adhesion molecules (*CNTN2*, *HLA‐DRB1*, *HLA‐DRB5*, and *NRXN3*). This indicates that it may be necessary to identify other dysregulated pathways to achieve therapeutic benefits.

We also compared the clinicopathological data of CRC patients with the mutational status of important signaling pathways in cancerous tissues. RTK‐RAS pathway mutations were correlated with tumor size (*P* = 0.028). These results suggest that tumor progression is not linked to increased genetic instability, although this may be due to our small sample size and fact that most cases were stage II (48.39% cases); we need to collect more samples to confirm our results.

In conclusion, we identified recurrent mutations in genes such as *APC*, *TP53*, *KRAS*, and *FBXW7*, as well as unreported mutations in *NRAS*, *PIK3CA*, *SOX9*, *APC*, *SMAD4*, *MSH3*, *MSH4*, *PMS1 PMS2*, *AXIN2*, *ERBB2*, *PIK3R1*, *TGFBR2*, and *ATM* in a group of Taiwanese CRC patients. The data presented herein provide more comprehensive characteristics of the top deadly disease and identify a possibility for treating it in a targeted way.
