# Supplementary material for: Molecular characterization of colorectal cancer using whole‐exome sequencing in a Taiwanese population
Source: Cancer Med. 2019 May 24;8(8):3738–47. doi: 10.1002/cam4.2282 (PMC6639182; doi:10.1002/cam4.2282)

**Supplementary Table 1.** List of discovered variants detected by WES in MMR gene wild-type carriers.

| **Patient ID** | **cancer-related gene (ClinVarAnnotation Pathogenic)** | **cancer-related gene (Pathogenicity predictions)** | **non-cancer-related gene (ClinVar Annotation Pathogenic)** | **non-cancer-related gene (Pathogenicity predictions)** | **Total** |
| --- | --- | --- | --- | --- | --- |
| 25 | 0 | 10 | 5 | 384 | 399 |
| 36 | 0 | 16 | 3 | 397 | 416 |
| 50 | 0 | 10 | 1 | 243 | 254 |
| 56 | 1 | 12 | 4 | 400 | 417 |
| 62 | 0 | 17 | 6 | 374 | 397 |
| 89 | 0 | 15 | 4 | 397 | 416 |
| 98 | 0 | 8 | 6 | 287 | 301 |
| 99 | 2 | 15 | 6 | 406 | 429 |
| 103 | 1 | 11 | 6 | 404 | 422 |
| CC01 | 1 | 13 | 4 | 299 | 317 |
| CC02 | 4 | 10 | 4 | 309 | 327 |
| CC03 | 2 | 10 | 4 | 330 | 346 |
| CC04 | 1 | 13 | 6 | 298 | 318 |
| CC06 | 3 | 11 | 3 | 298 | 315 |
| CC07 | 2 | 11 | 3 | 325 | 341 |
| CC10 | 2 | 20 | 5 | 378 | 405 |
| CC11 | 2 | 13 | 3 | 380 | 398 |
| CC12 | 1 | 15 | 3 | 329 | 348 |
| CC13 | 0 | 10 | 3 | 392 | 405 |
| CC14 | 1 | 11 | 0 | 377 | 389 |
| CC15 | 1 | 18 | 2 | 371 | 392 |
| CC16 | 2 | 11 | 3 | 325 | 341 |
| CC17 | 2 | 11 | 5 | 298 | 316 |
| CC18 | 3 | 8 | 3 | 307 | 321 |
| CC20 | 1 | 13 | 2 | 298 | 314 |
| CC21 | 1 | 10 | 0 | 293 | 304 |
| CC24 | 0 | 11 | 5 | 270 | 286 |

**Supplementary Table 2.** List of discovered variants detected by WES in MMR gene mutation carriers.

| **Patient ID** | **Mutation of MMR genes** | **Mutatuon type^a^** | **Cancer-related gene (ClinVarAnnotation Pathogenic)** | **Cancer-related gene (Pathogenicity predictions)** | **Non-cancer-related gene (ClinVar Annotation Pathogenic)** | **Non-cancer-related gene (Pathogenicity predictions)** | **Total** |
| --- | --- | --- | --- | --- | --- | --- | --- |
| CC-08 | *MSH3* p.E456K | Novel | 4 | 89 | 14 | 1376 | 1483 |
|  | *PMS1* p.R265Q | Novel |  |  |  |  |  |
|  | *PMS2* p.L633I | Novel |  |  |  |  |  |
| 16 | *MSH4* p.E583* | Novel | 3 | 87 | 12 | 1109 | 1211 |
| 93 | *MLH1* p.T117M | Known | 1 | 30 | 7 | 472 | 510 |
| 71 | *MSH3* p.A61delinsAAPA | Novel | 0 | 14 | 0 | 321 | 335 |
| CC-05 | *MLH1* p.R385C | Known | 2 | 8 | 4 | 315 | 329 |

^a^Mutation type is novel if no dbSNP, COSMIC and TCGA entry were found.

**Supplementary Figure 1.** WES reads showing mutation (A) *NRAS* c.203C>A p.Q61H (B) *PIK3CA* c.1048G>T p.D350Y (C) *SOX9* c.1452_1456del p.S484fs and c.1453_1454del p.G485fs (D) *APC* c.1620dupA p.L540fs, *APC* c.3875_3876insA p.T1292fs, *APC* c.3889_3905del p.D1297fs, *APC* c.3904_3905insA p.L1302fs, *APC* c.3917delA p.E1306fs, *APC* c.4122_4129del p.E1374fs, *APC* c.5746C>T p.Q1916* (E) *SMAD4* c.779dupA p.Y260_H261delins* (F) *MSH3* c.183_184insGCGCCCGCC p.A61delinsAAPA and c.1366G>A p.E456K (G) *MSH4* c.1747G>T p.E583* (H) *PMS1* c.794G>A p.R265Q (I) *PMS2* c.1897C>A p.L633I (J) *AXIN2* c.1376G>T p.R459L (K) *ERBB2* c.26dupG p.W9fs (L) *PIK3R1* c.440delC p.S147* and c.482T>G p.L161* (M) *TGFBR2* c.1646A>C p.D549A (N) *ATM* c.1948G>T p.E650*.

(A) *NRAS* c.203C>A p.Q61H


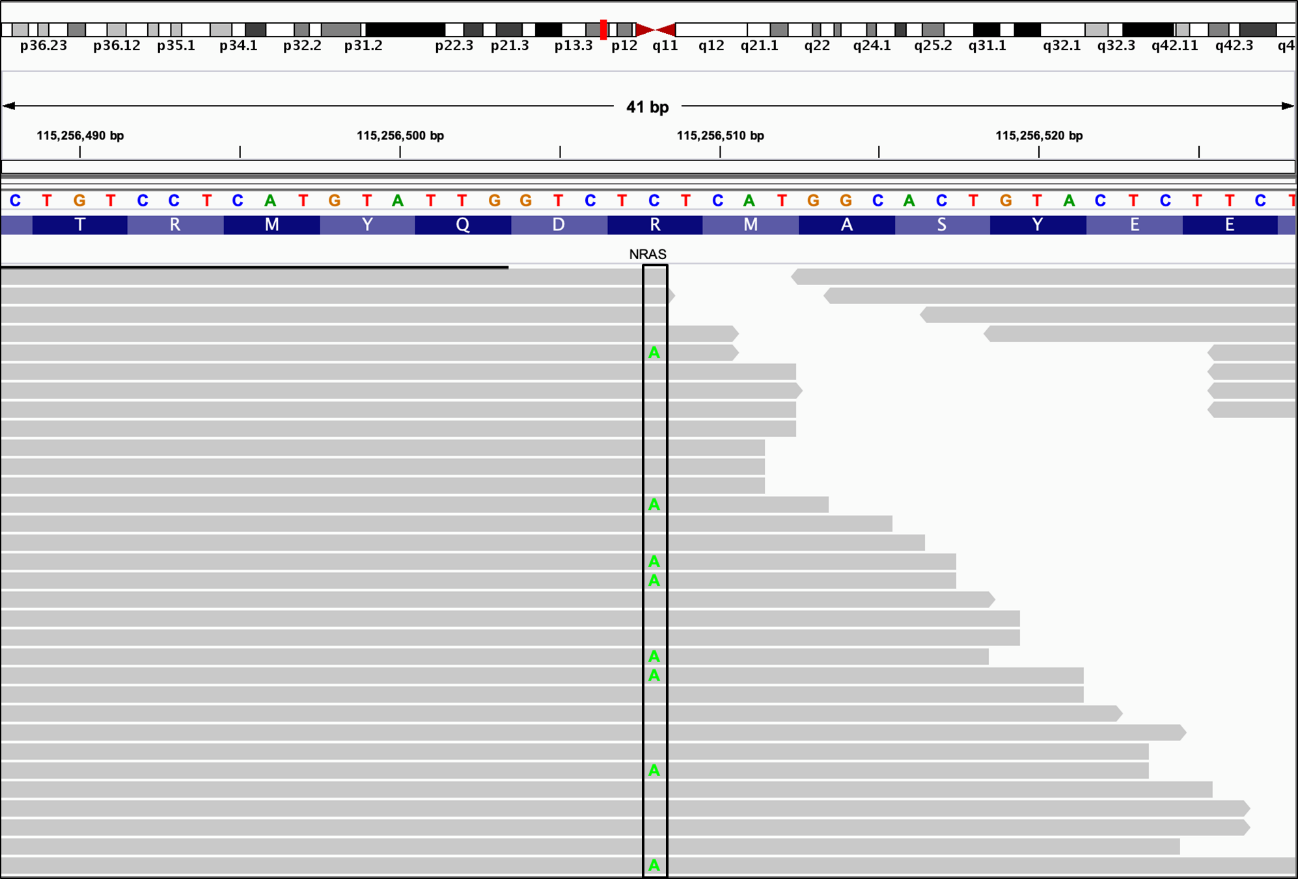


(B) *PIK3CA* c.1048G>T p.D350Y


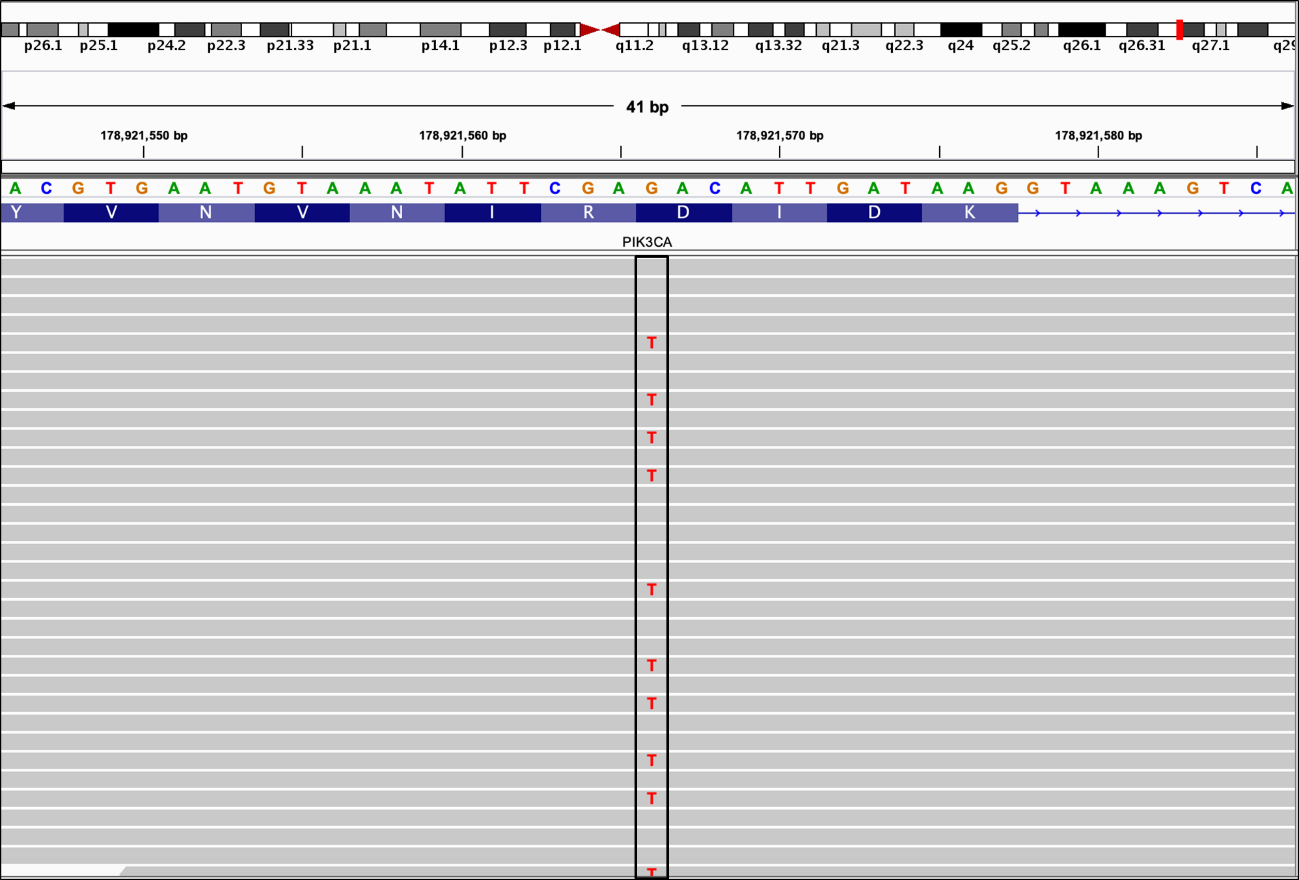


(C) *SOX9* c.1452_1456del p.S484fs

*
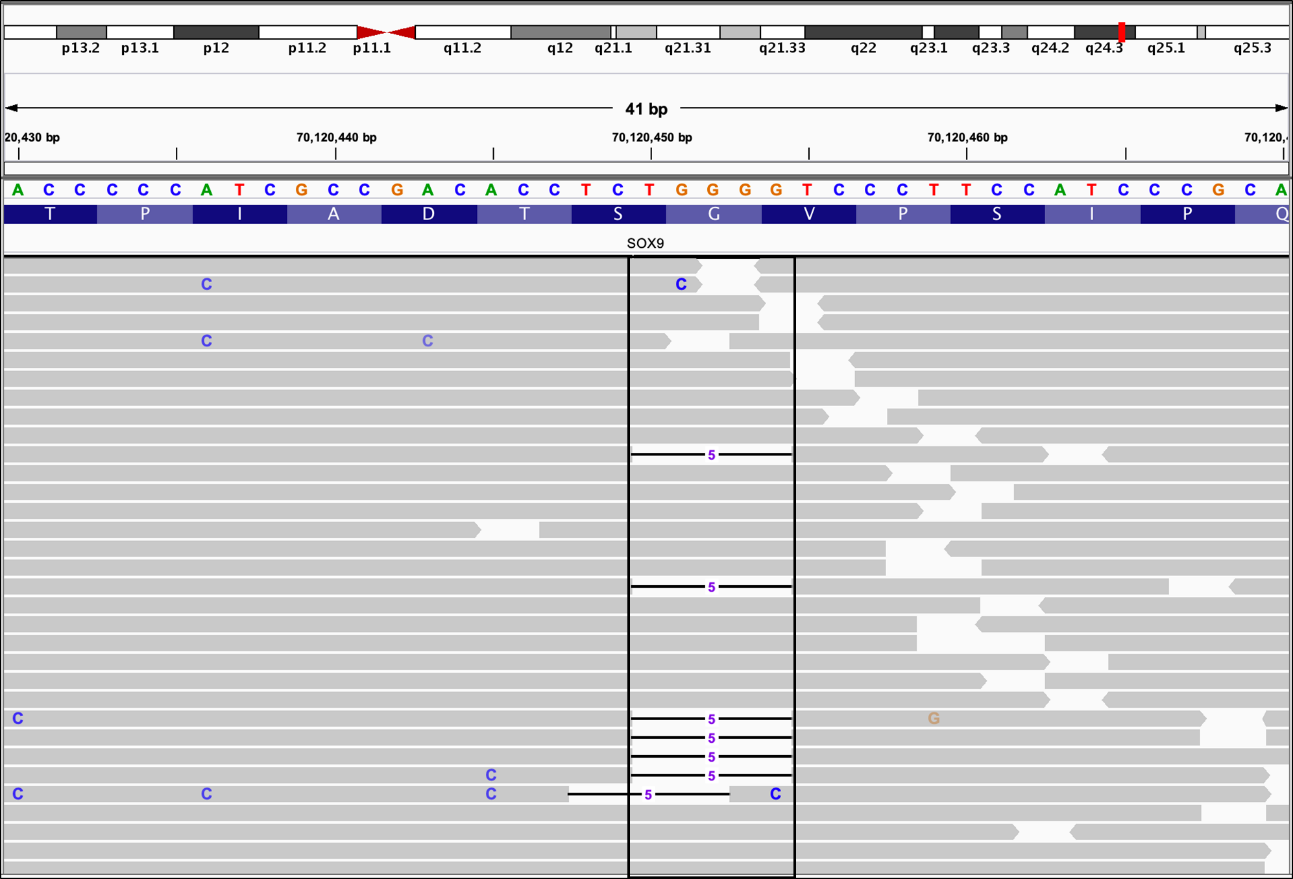
*

*SOX9* c.1453_1454del p.G485fs


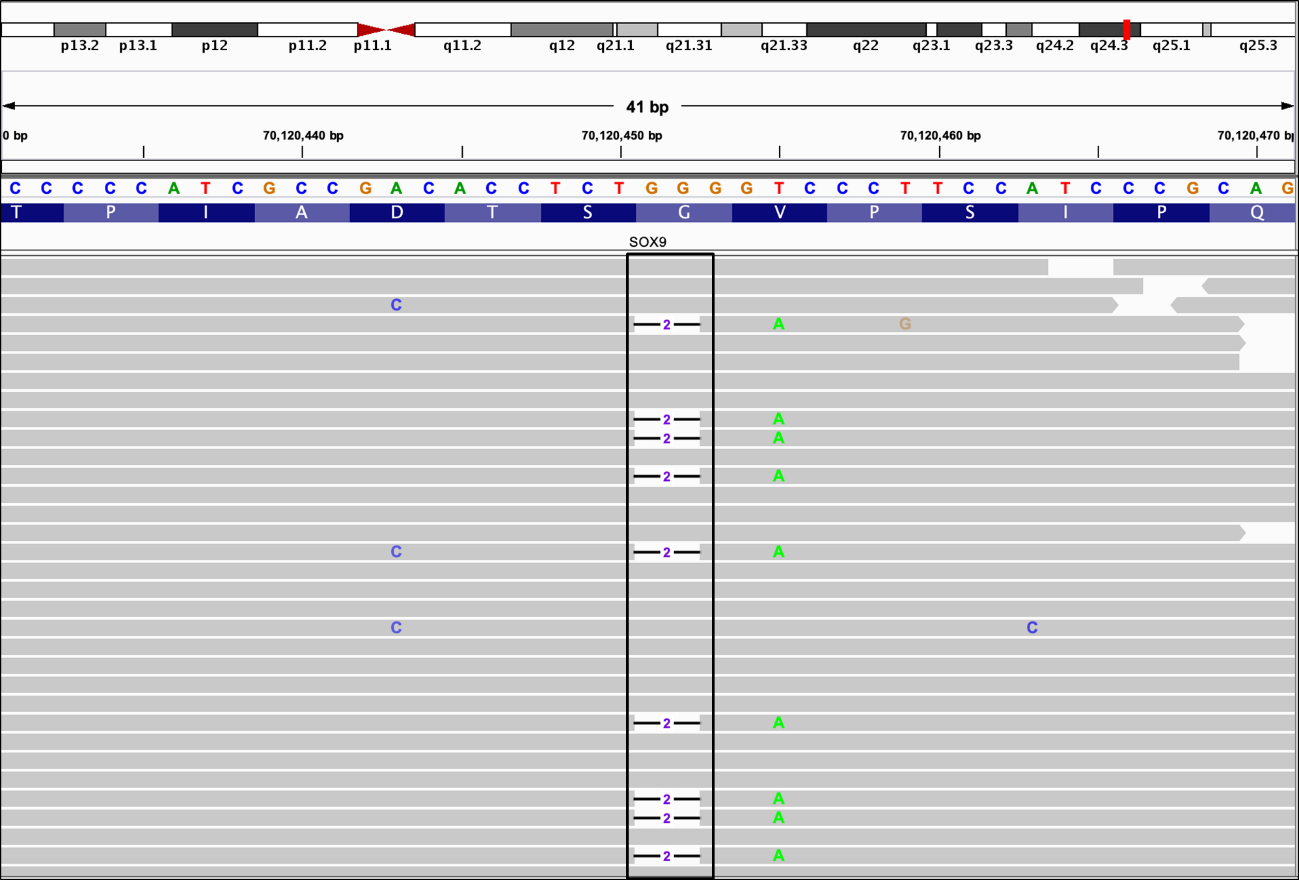


(D) *APC* c.1620dupA p.L540fs


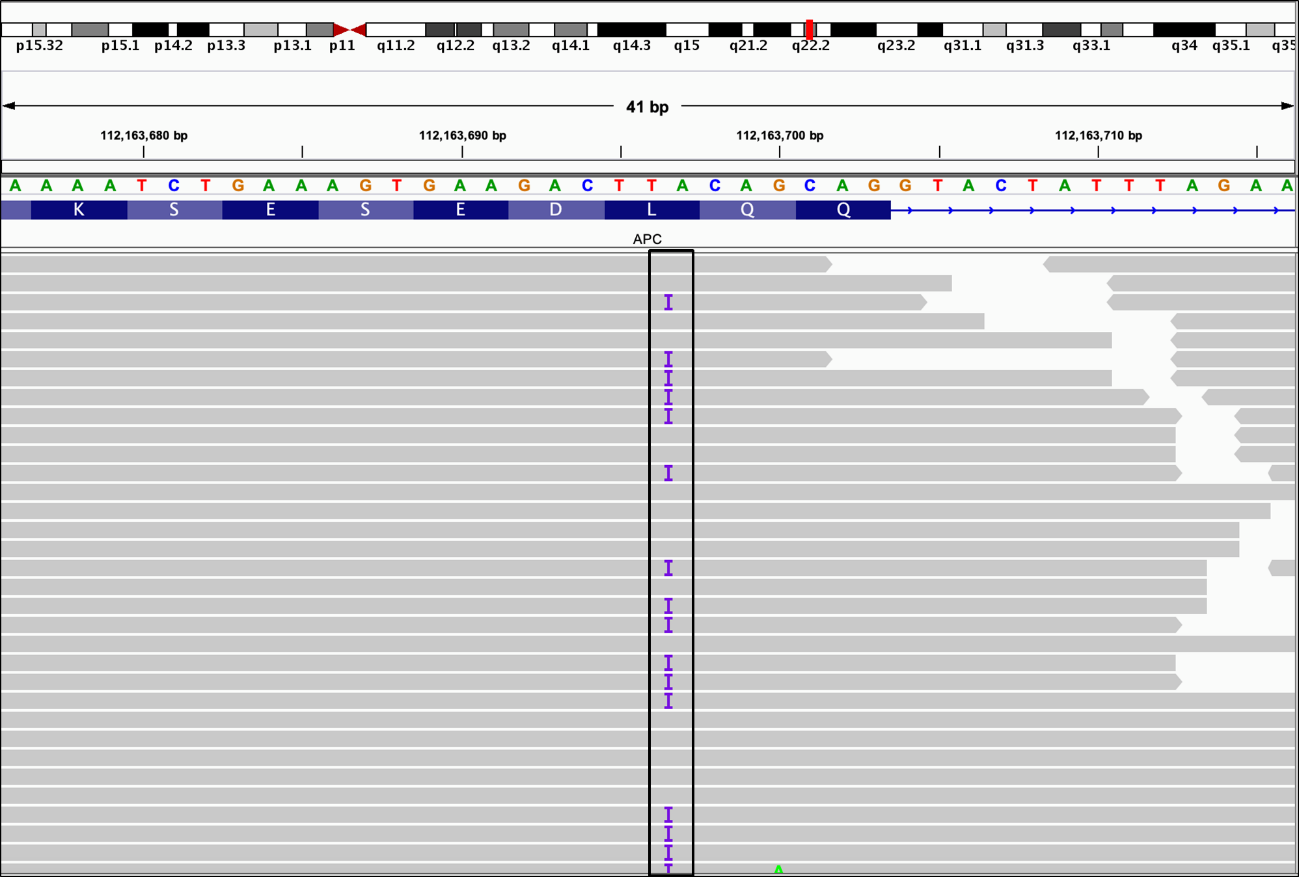


*APC* c.3875_3876insA p.T1292fs


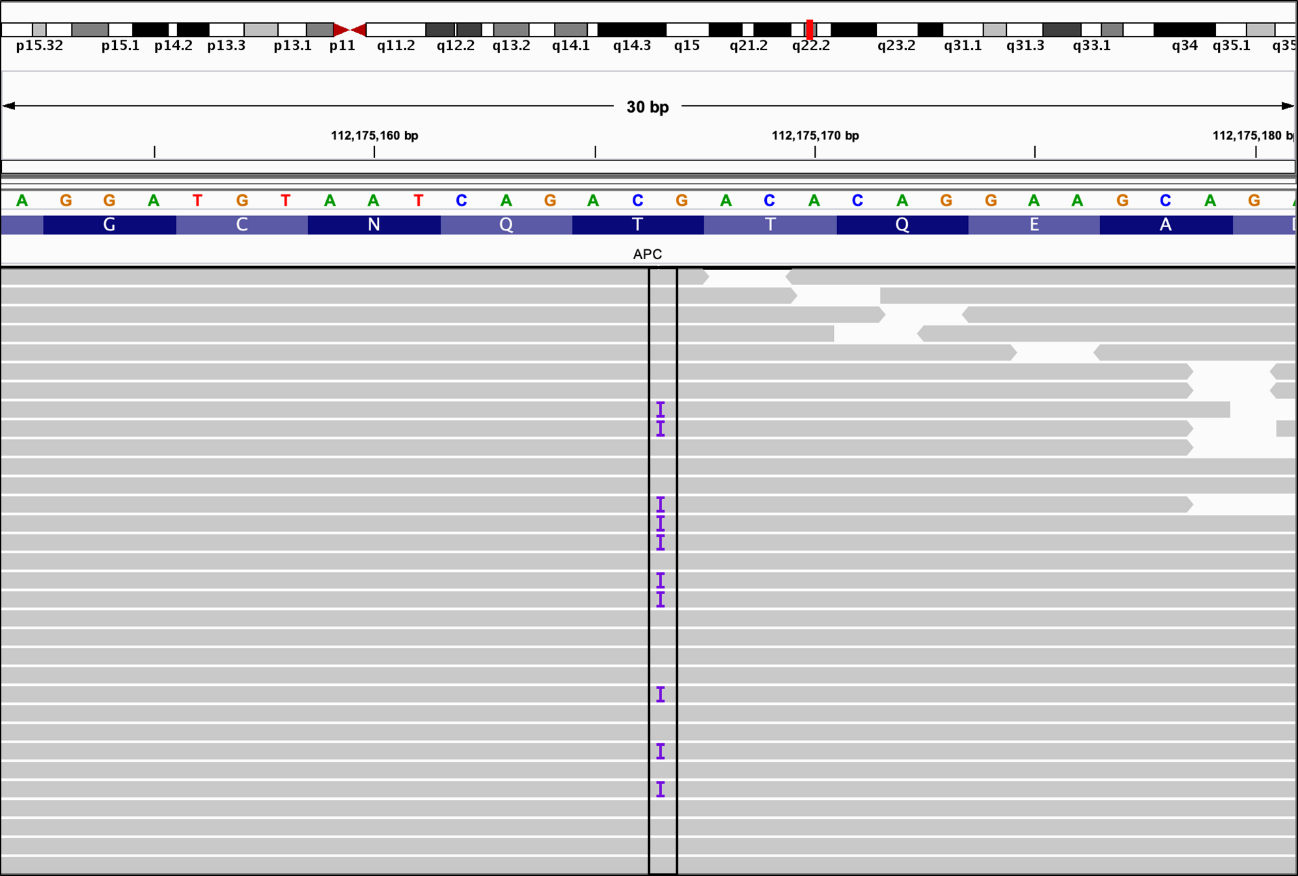


*APC* c.3889_3905del p.D1297fs


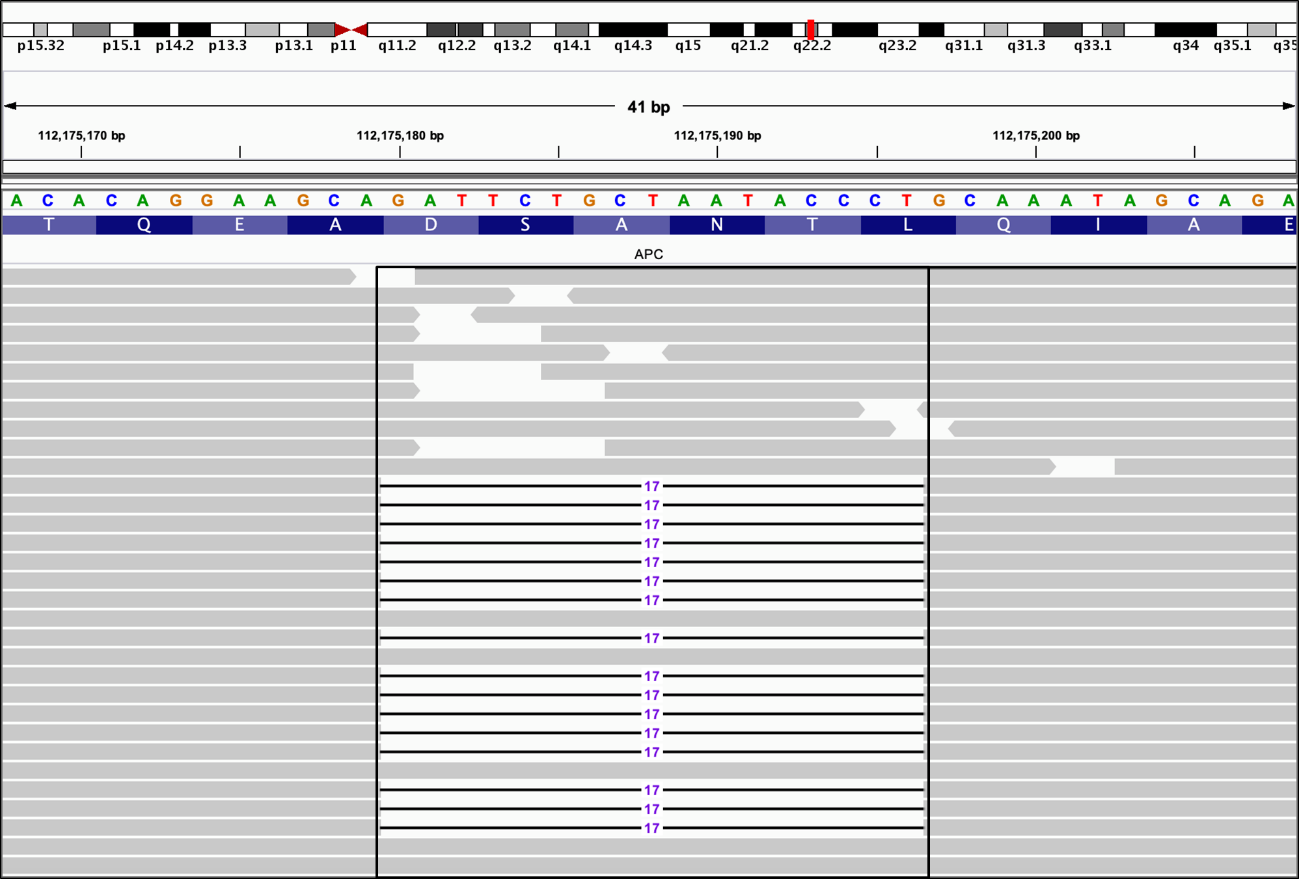


*APC* c.3904_3905insA p.L1302fs


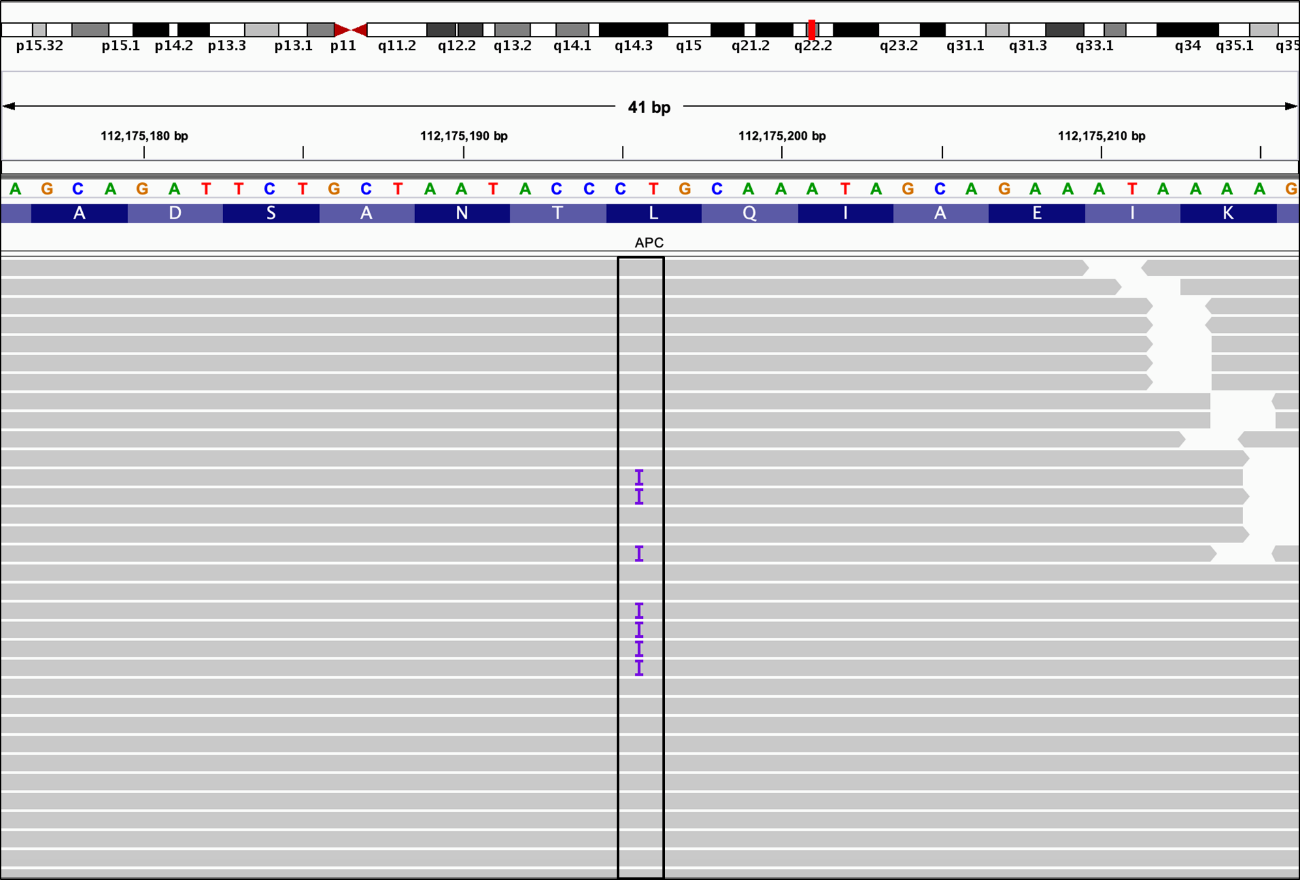


*APC* c.3917delA p.E1306fs


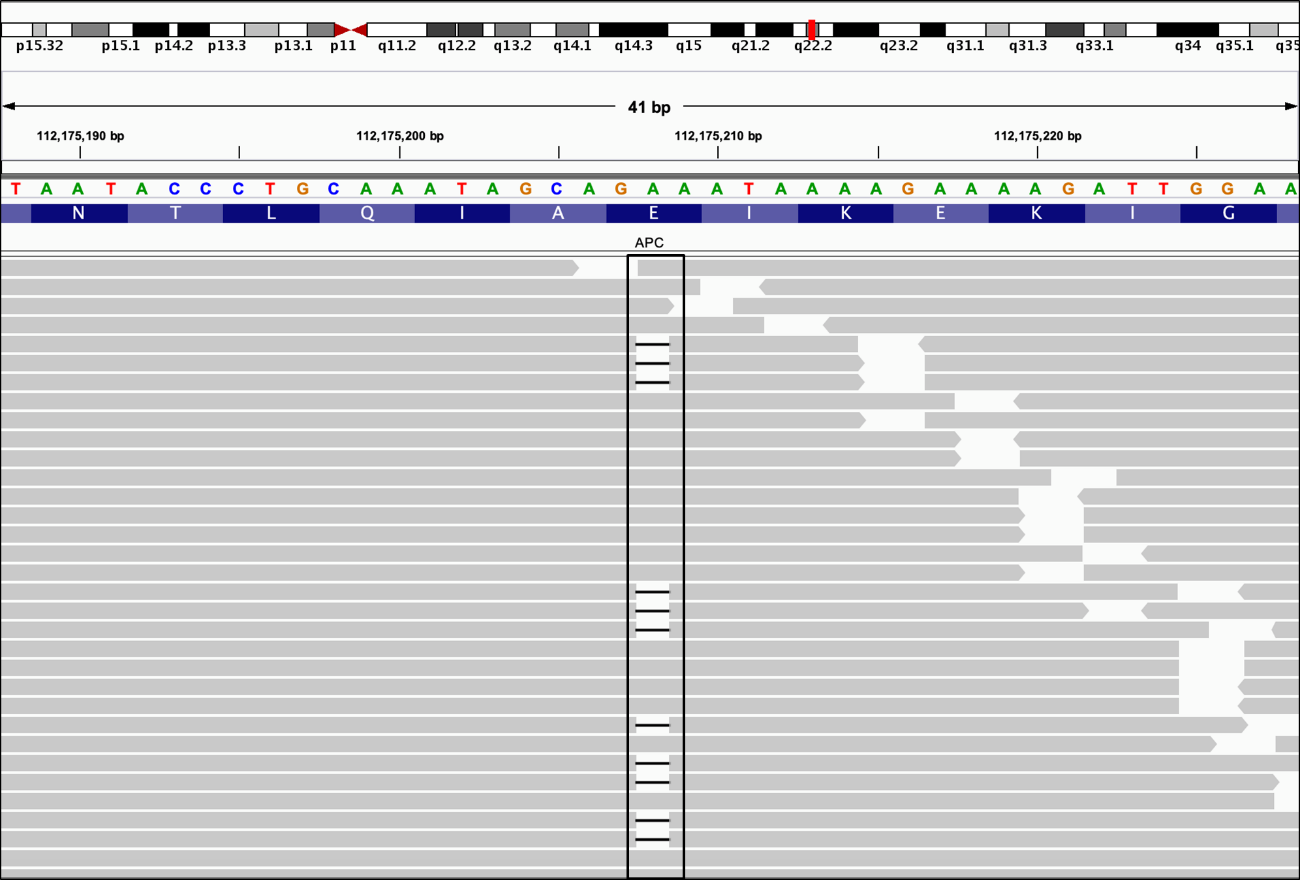


APC c.4122_4129del p.E1374fs


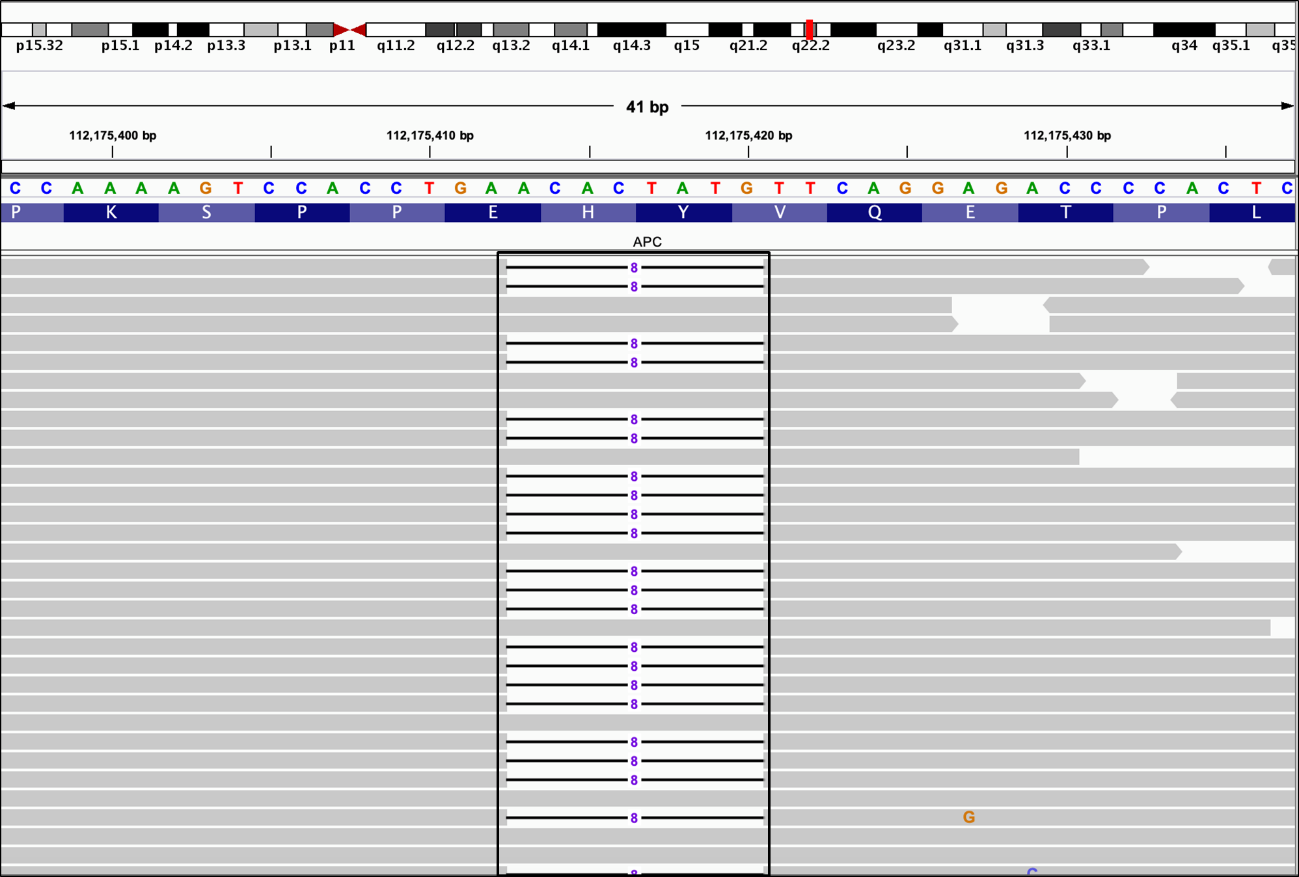


*APC* c.5746C>T p.Q1916*


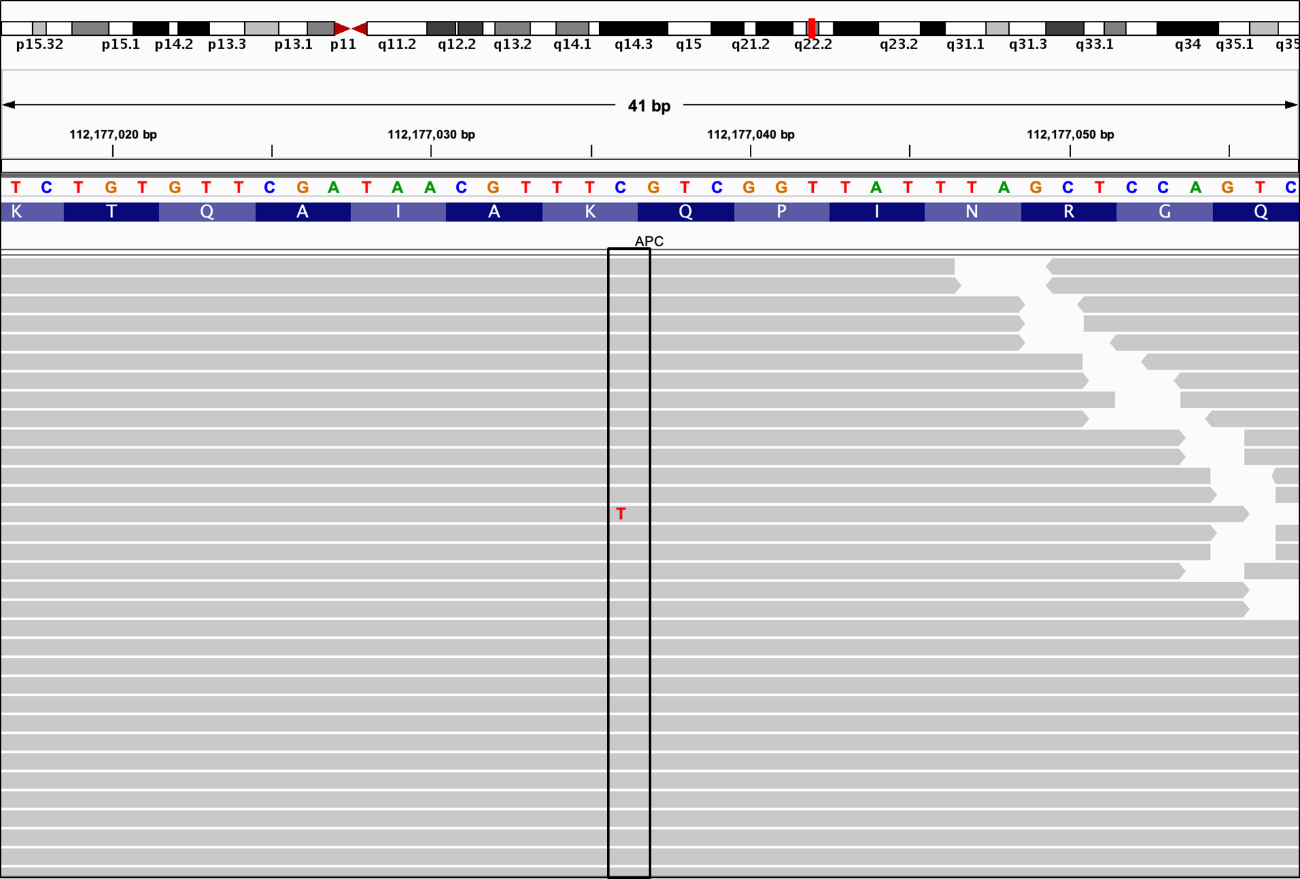


(E) *SMAD4* c.779dupA p.Y260_H261delins*


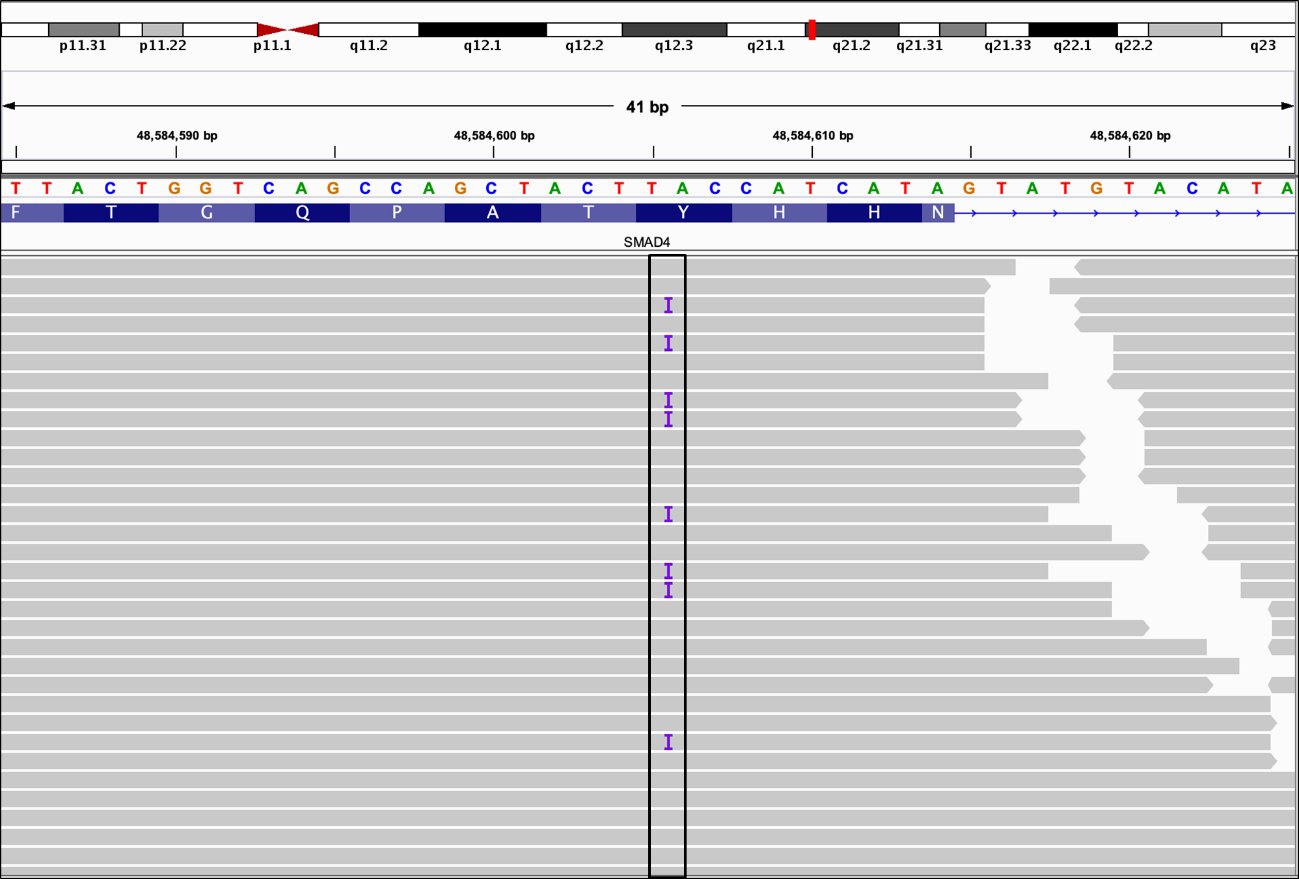


(F) *MSH3* c.183_184insGCGCCCGCC p.A61delinsAAPA

*
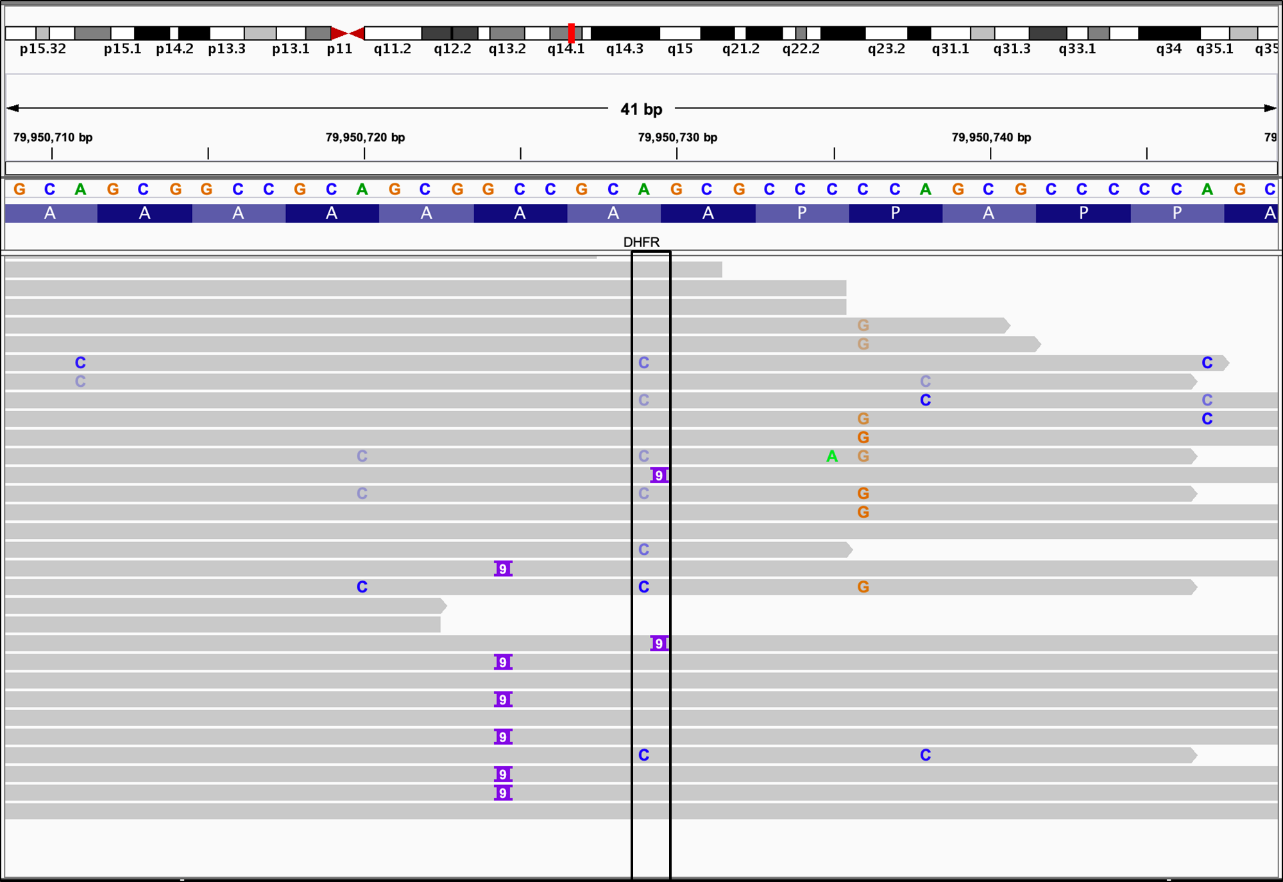
*

*MSH3* c.1366G>A p.E456K


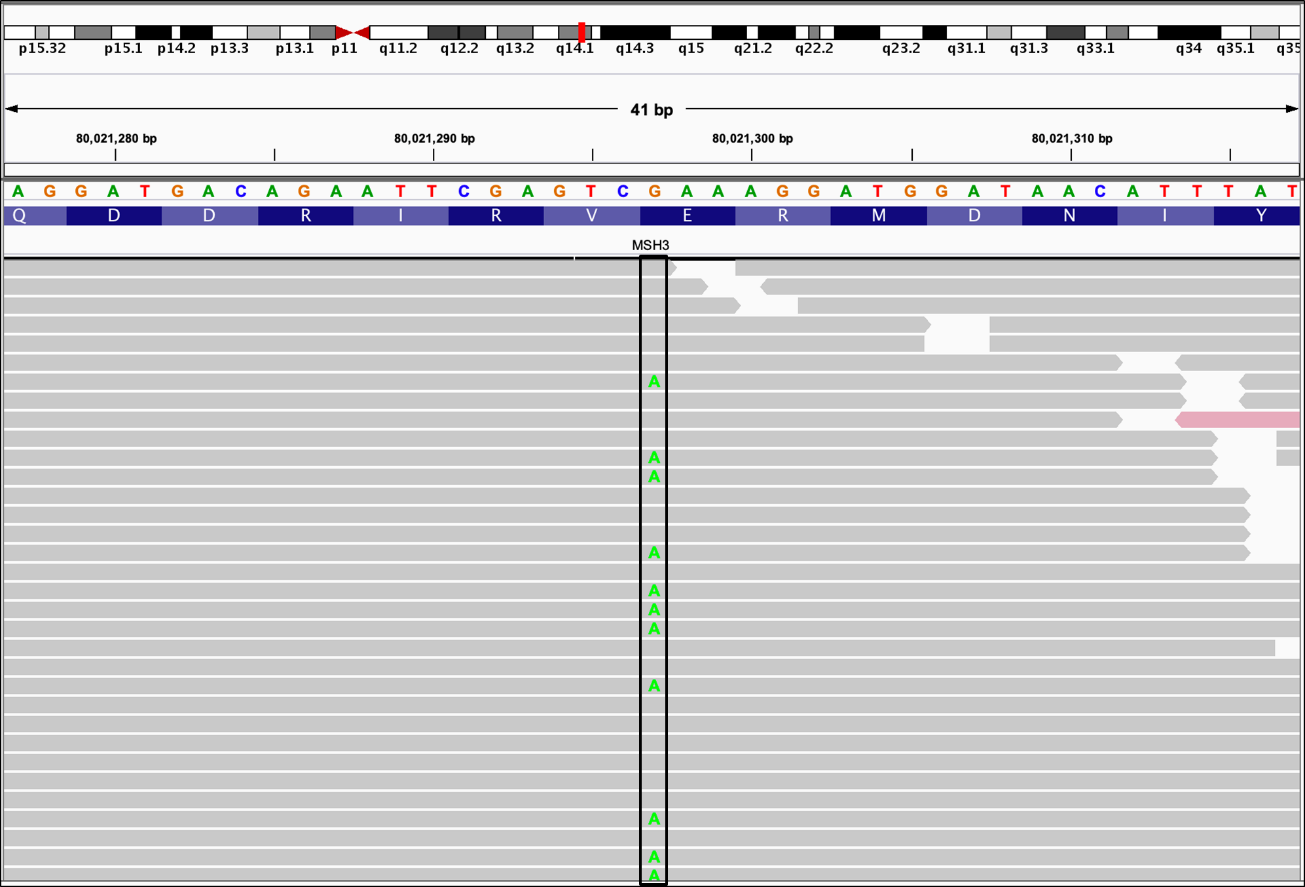


(G) *MSH4* c.1747G>T p.E583*


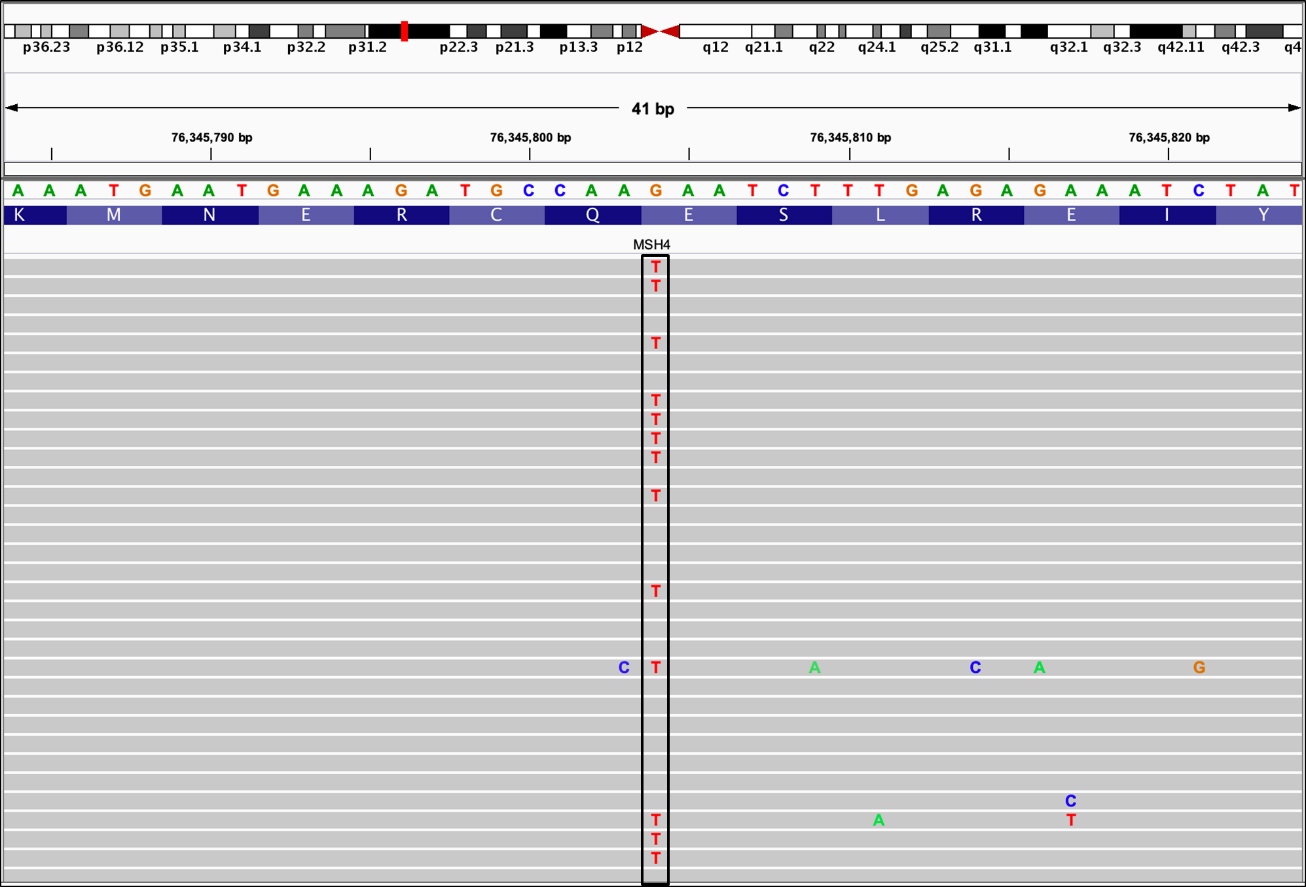


(H) *PMS1* c.794G>A p.R265Q


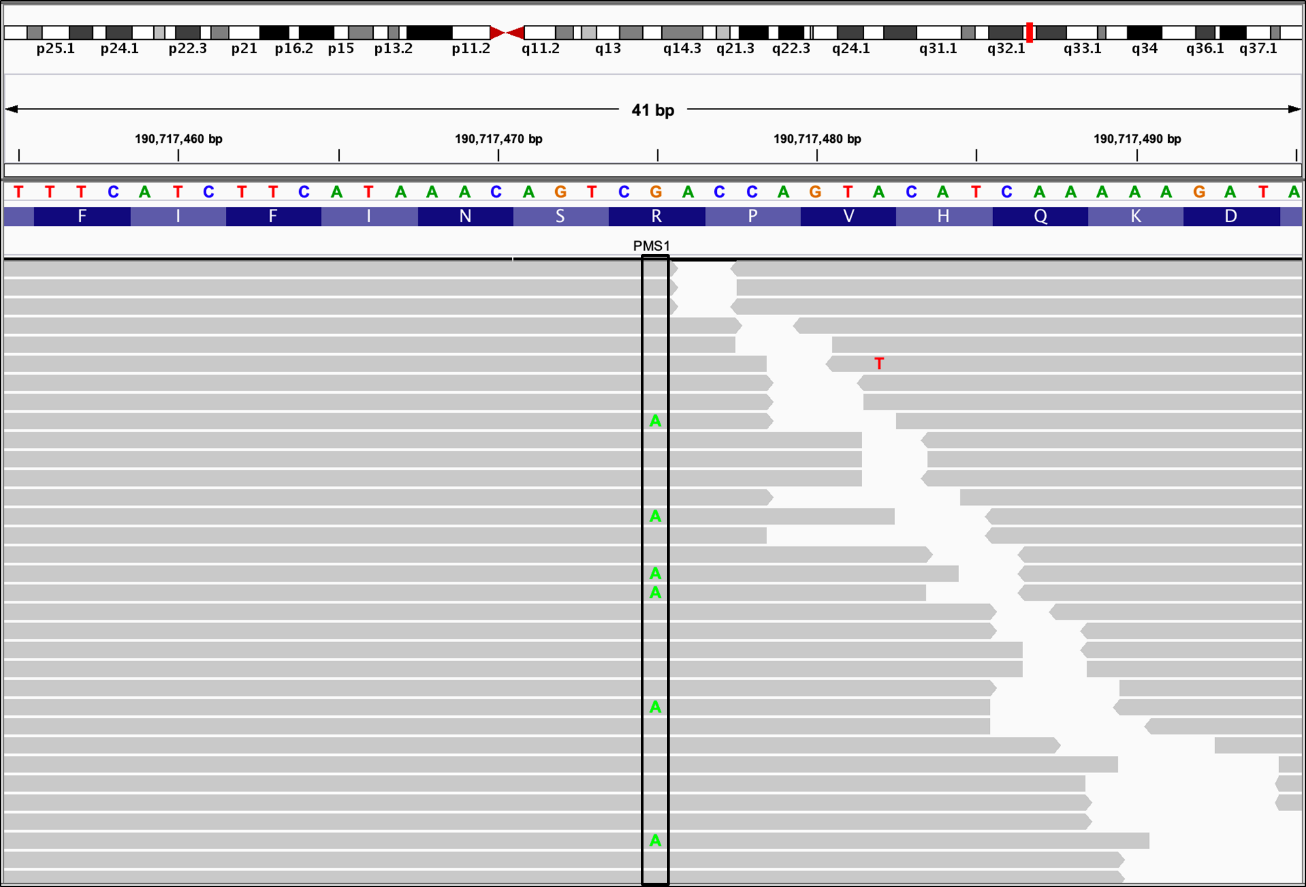


(I)*PMS2* c.1897C>A p.L633I


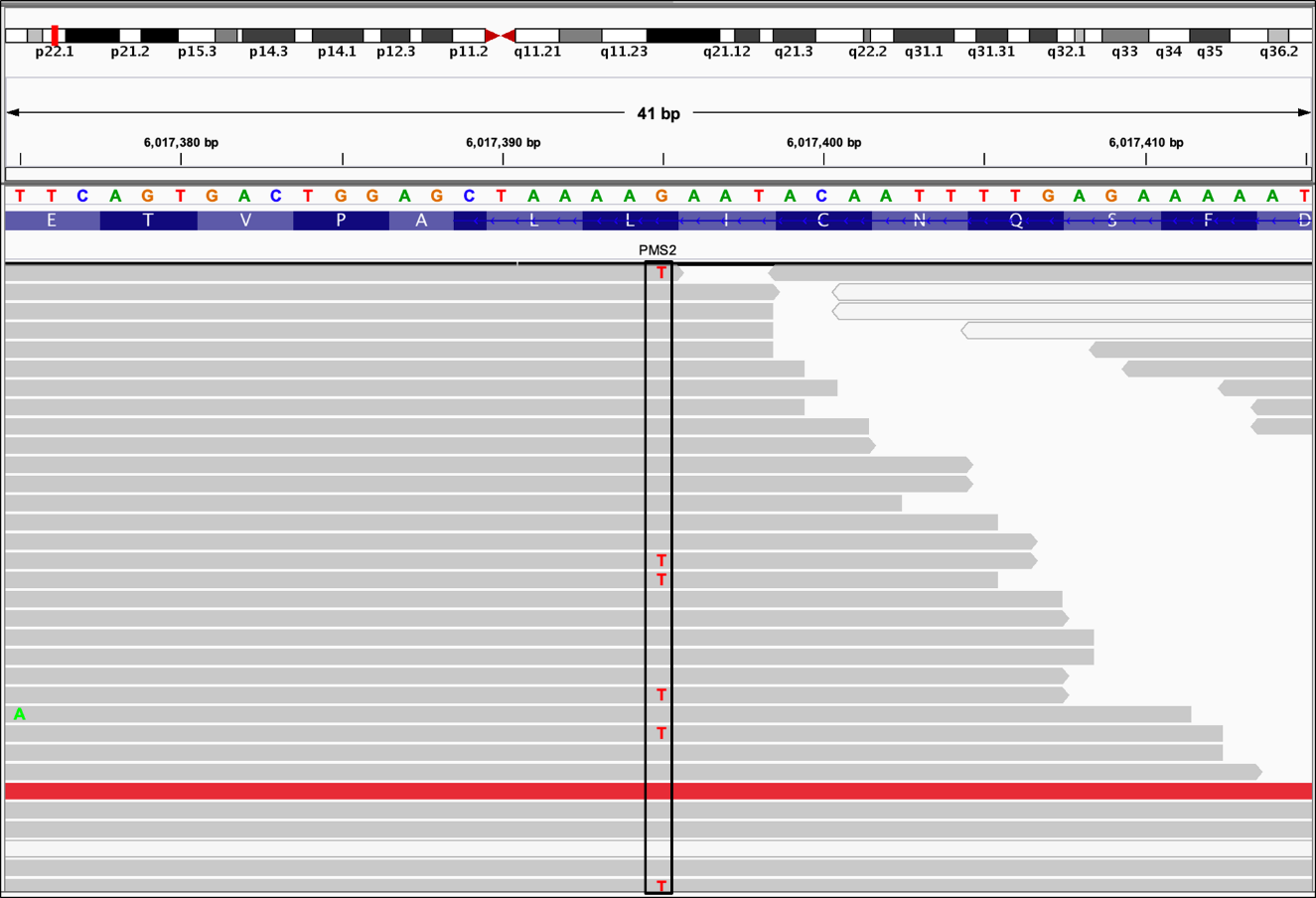


(J) *AXIN2* c.1376G>T p.R459L


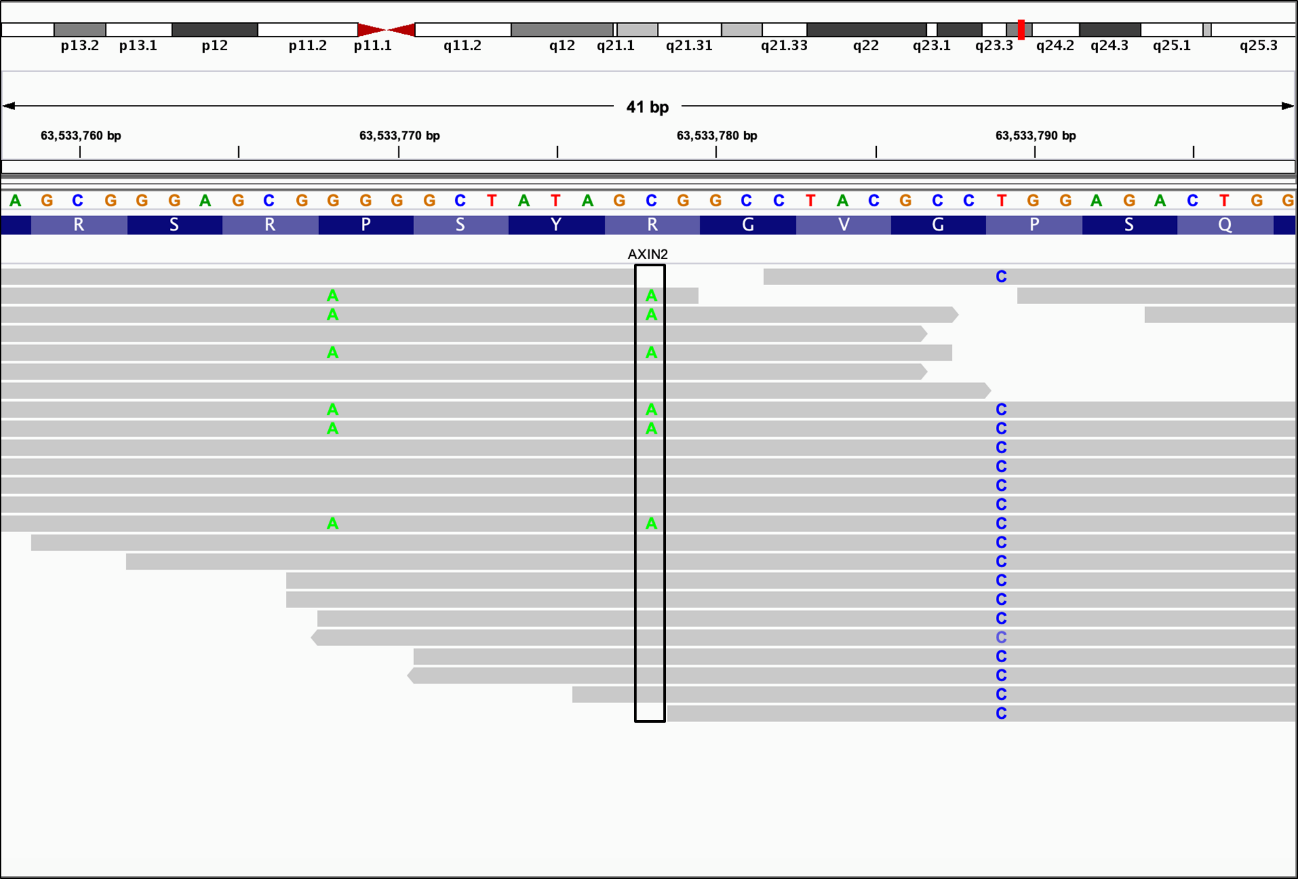


(K) *ERBB2* c.26dupG p.W9fs


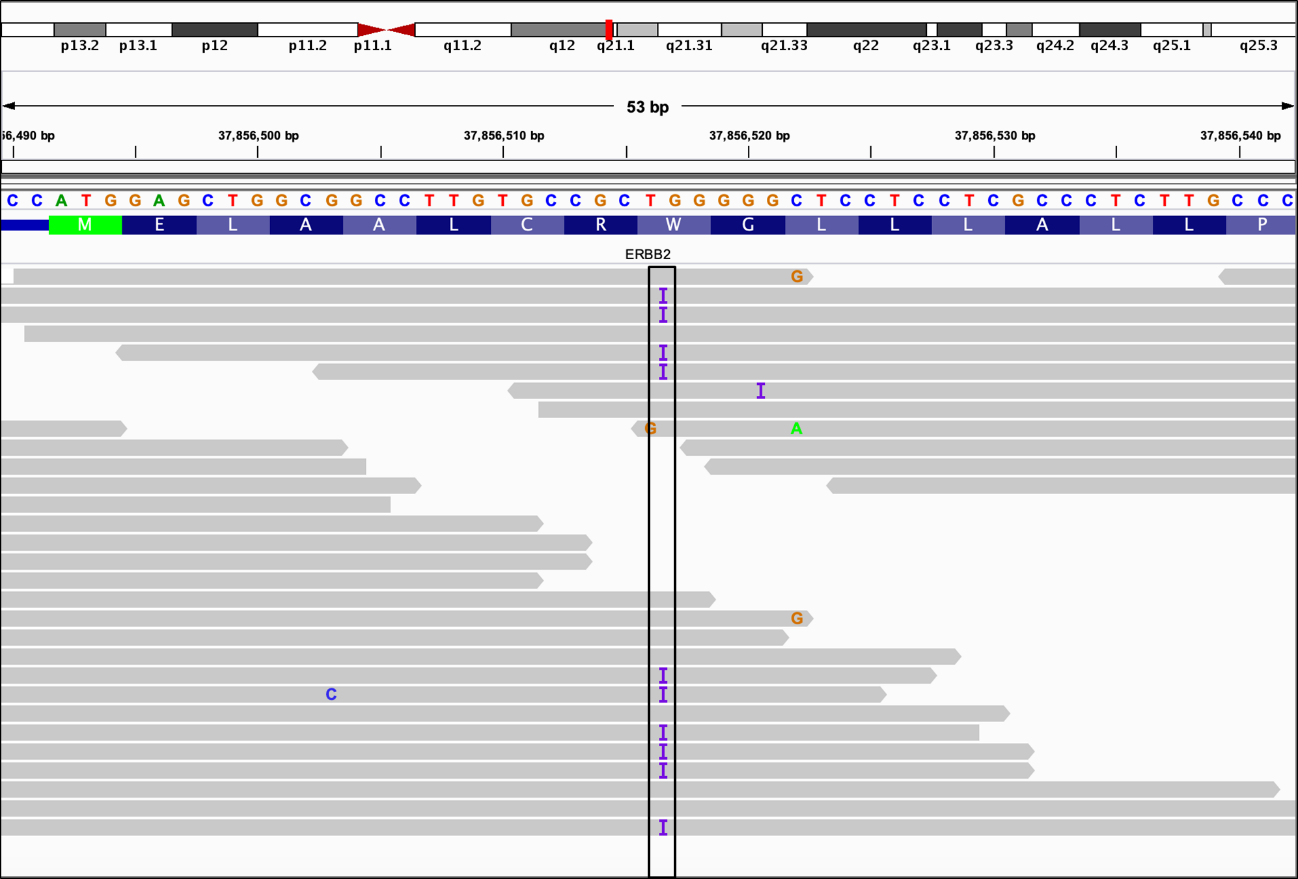


(L) *PIK3R1* c.440delC p.S147*


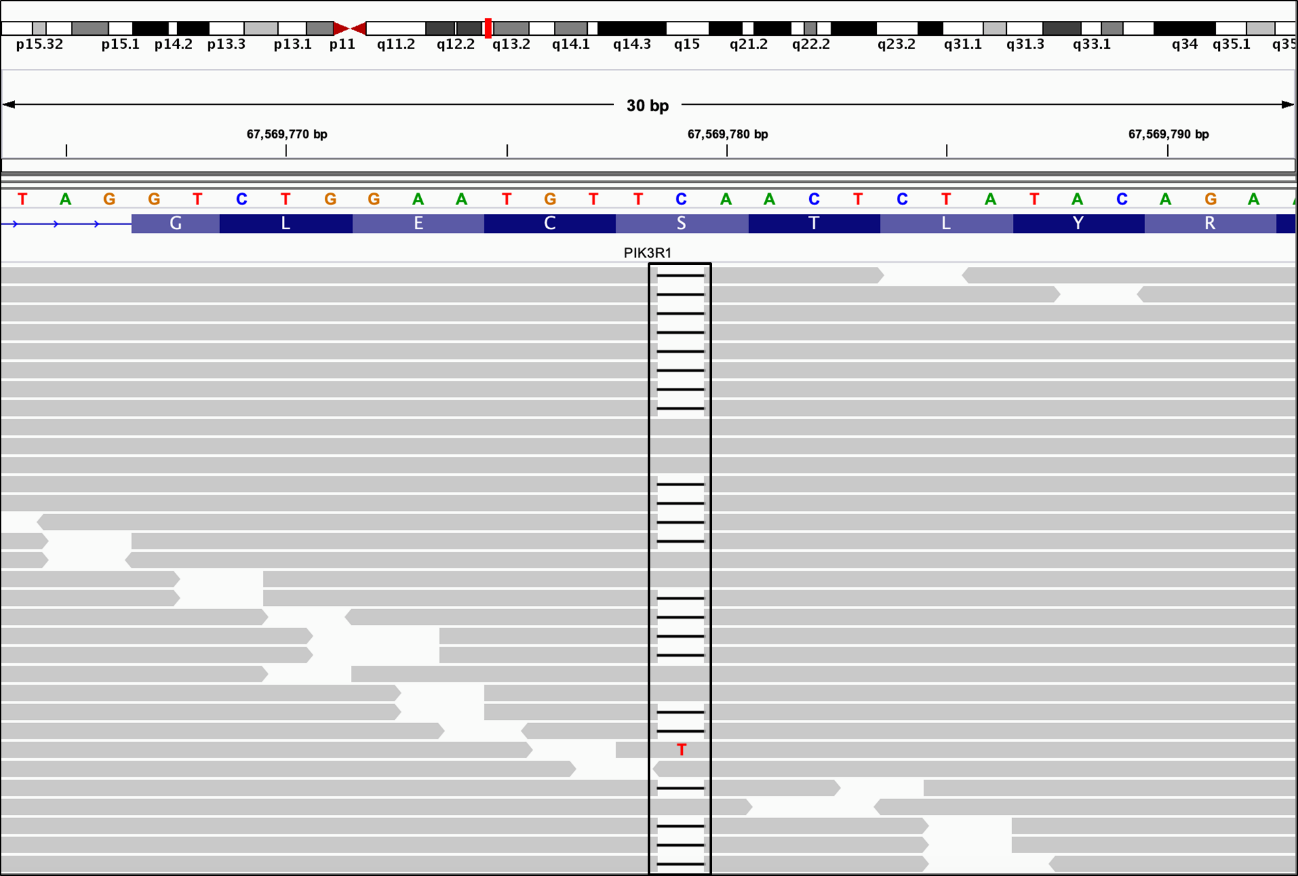


c.482T>G p.L161*


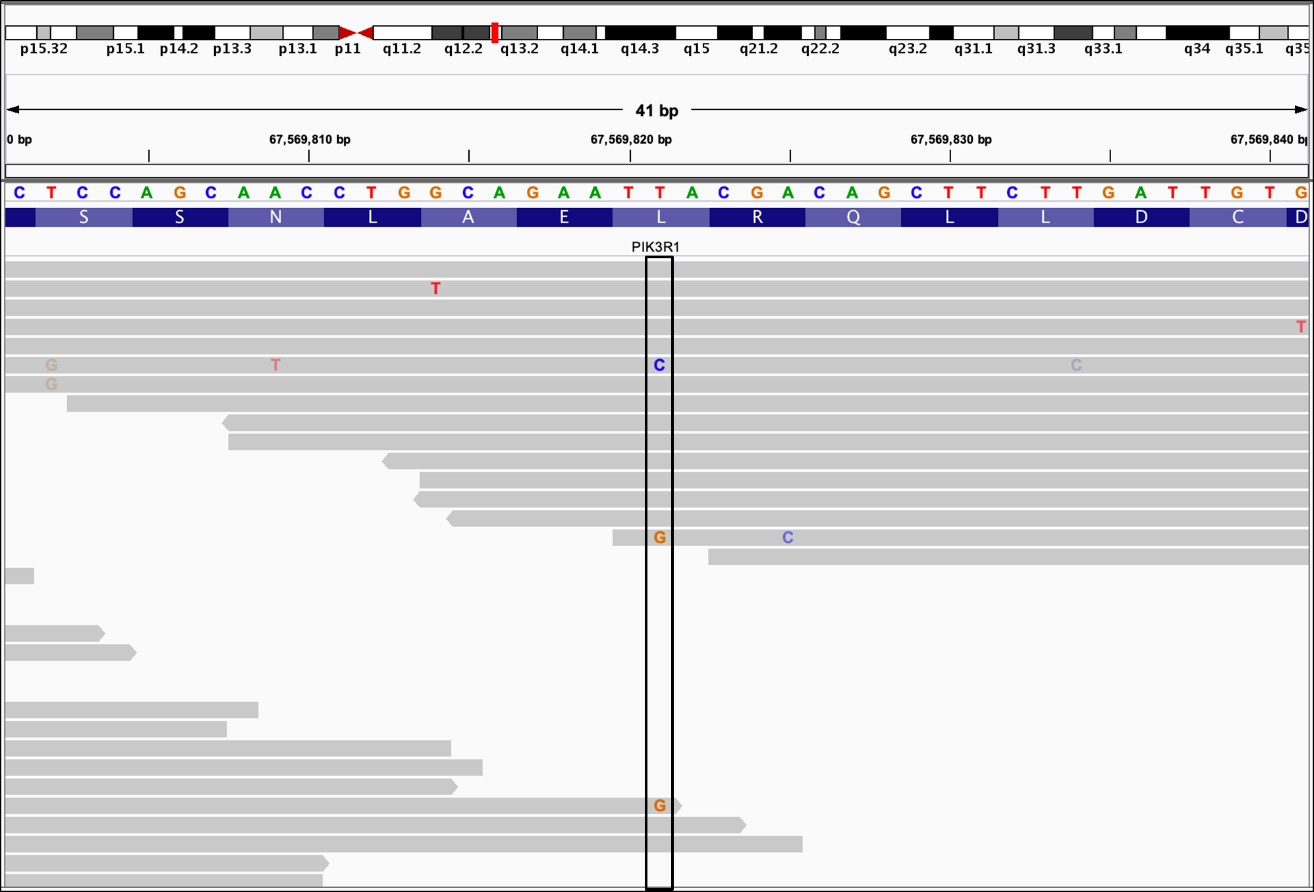


(M) *TGFBR2* c.1646A>C p.D549A


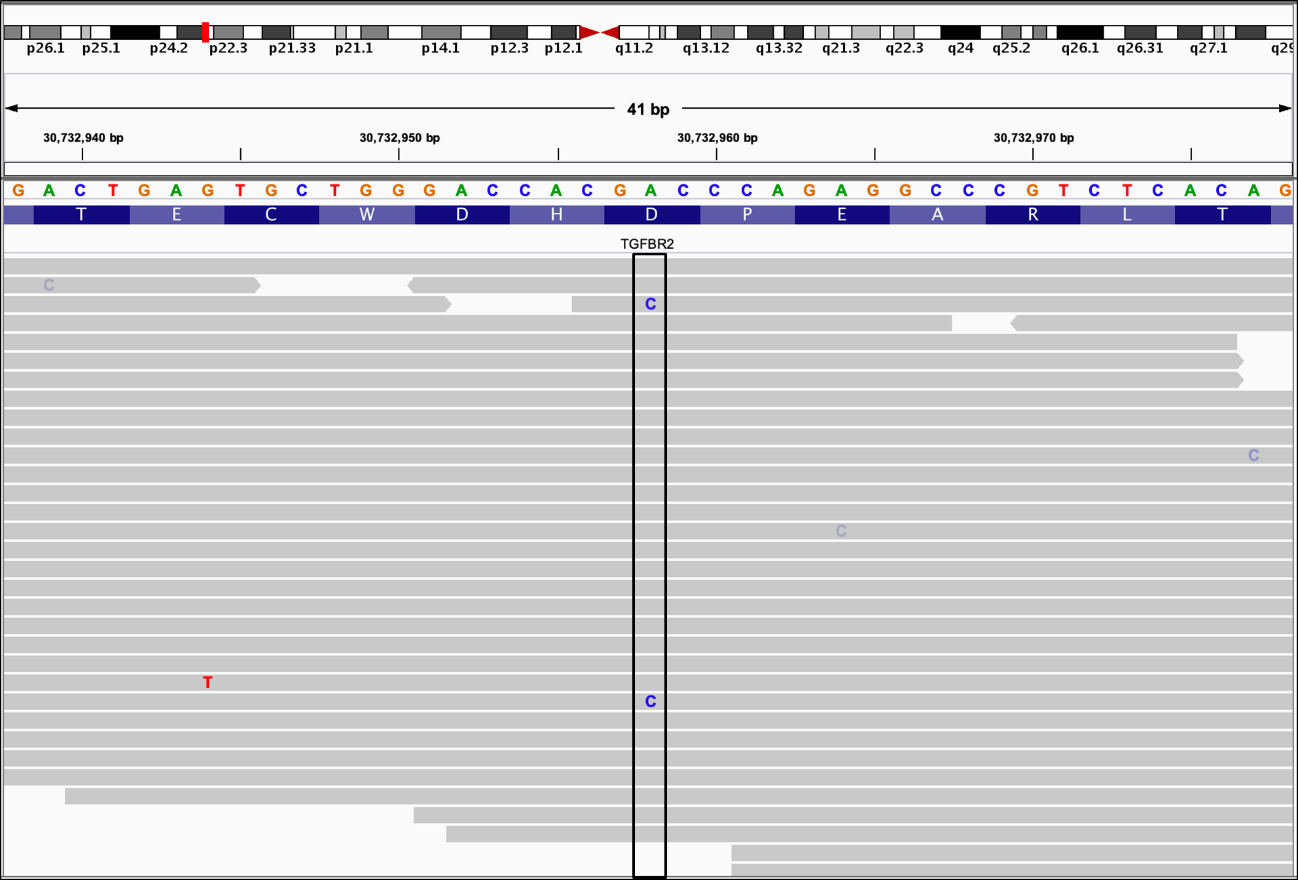


(N) *ATM* c.1948G>T p.E650*


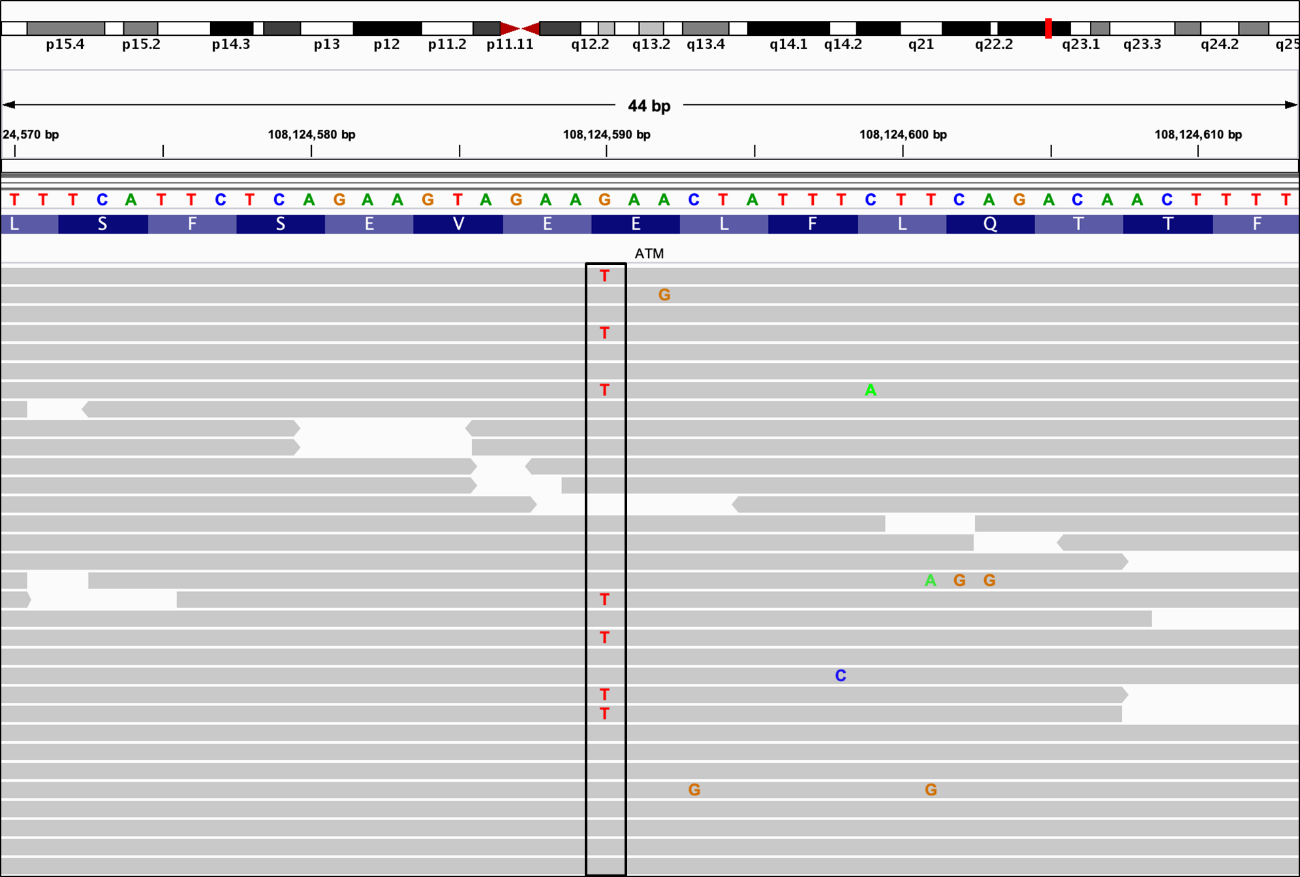

Supplement: Supplementary file 1 [file CAM4-8-3738-s001.docx]
